# Interpol review of fingermarks and other body impressions 2019 – 2022)

**DOI:** 10.1016/j.fsisyn.2022.100304

**Published:** 2022-12-28

**Authors:** Andy Bécue, Christophe Champod

**Affiliations:** University of Lausanne, School of Criminal Justice, Faculty of Law Criminal Justice and Public Administration, Switzerland

## 1 Introduction

The area of fingermarks is a very active community fuelled with many research papers, case studies and commentaries proposed during the reviewing period (July 2019 – June 2022). We have attempted to cover the main developments focusing on mainstream peer-reviewed journals. With 630 references collected, for ca. 500 cited in this paper, we observe a slight increase compared to the previous reviewing period that counted 599 references [1], which confirms the strong interest for the discipline by the research community. Roberts et al. [2] gave similar evidence of that interest.

At this outset of this report, we would like to draw attention to a few monographs that are covering various topics discussed in this review:

– Perkins [3] provides an highly documented, accessible and practical introduction to the ACE framework fingerprint examiners use to analyze, compare and evaluate friction ridge skin impressions.
– Daluz Moses [4] published a book devoted to the presentation of fingerprint evidence in court.
– Hawthorne, Plotkin and Douglas [5] published their second edition of “Fingerprints”, a broad introduction to the field.
– Scarborough, Henning and Leo [6] published a vivid response to what they name an attack or hijack of the discipline by critics, academics and other scholars.

We also would like to draw the attention on the forthcoming 3rd edition of the Encyclopaedia of Forensic Sciences [7] that offers numerous chapters associated with fingerprint detection and identification.

The area of fingerprint biometry (and other types of body impressions) is too wide and active to allow a comprehensive review. For this contribution, we have favoured papers that illustrate new trends in either the features considered (e.g. level 3 features or incipient ridges) or new types of algorithms with an application to marks. We remain conscious that this represents only a snapshot of the research activity in the biometric field. For a complete overview we invite to refer to the third edition of the handbook of fingerprint recognition [8] and a recent review paper [9]. Deep learning approaches currently outperform other approaches (e.g. feature crafted) and gained significant attention in the research community.

## 2 Friction ridge skin and its individualization process

### 2.1 Research associated with friction ridge skin features

In introduction to this section we would like to highlight the review paper by Alice White [10] that presents the various features of the friction ridge skin, the attributes, their expected usefulness for establishing search parameters and in establishing identity, and the sources of variation in their appearance.

In addition, the paper by Monson et al. [11] counts among the most up-to-date review paper and research results on the permanence and persistence of friction ridge skin (when observed directly on the skin) and persistence of friction ridge impressions (when observed on prints or marks). Level 1 detail was reported as permanent and persistent. Persistence, but not permanence, was supported for level 2 details with small changes observed only in appearance. There were no changes in the presence of new, or absence of existing, minutiae. Level 3 details of ridge edge shape and pore presence were neither permanent nor persistent. The same was observed for incipient ridges, which were neither permanent nor persistent.

From infancy to adulthood, fingertip growth is anisotropic, much more along the proximal–distal axis than along the medial–lateral axis. A growth model has been published by Markert et al. [12]. It can help to compensate for growth and search prints in AFIS databases.

#### 2.1.1 Level 1

The use of ridge density (RD) to predict sex, age or ethnicity is a field with a remarkable activity (see Ref. [13] for a review). RD finds application in forensic science but also in archaeology studies [14,15]. Data regarding the difference between men and women fingerprints have been published in relation to a Brazilian population (200 individuals in total) [16]. The study confirmed that women have higher ridge densities compared to men. Significant differences in RDs between hands and fingers were also observed. However, the predictive power remains limited. Typically. the largest likelihood ratio is of the order of 20 for fingerprints. In Ref. [17], similar observations were made on a population of 150 male and 150 female Filipinos respectively. Note that the predictive capability is reduced when dealing with marks left under uncontrolled conditions. The same holds for the use of hand morphology to predict the sex of the donor [18].

The study of ridge widths between fingermarks and prints has shown that there are morphological changes over time [19]. Indeed, ridges widths on fingermarks tends to widen with aging compared to both rolled and flat impressions. A study on 768 fingermarks, left by one male and one female donors on varying substrates and subsequently visualized using different detection techniques, has shown that this ridge drift is conditioned by age but also depends on the substrate and detection technique [20].

Sex determination can also be helped with the measure of the fingerprint white line count (FWLC) or creases [[21], [22], [23], [24]]. FWLC exhibited leftward asymmetry in all the digits in both males and females and allows reasonable prediction of the sex of the donor. The same holds when white lines (creases) on palms are used on palm prints from a Malaysian population [25] or the palmar tri-radii [26].

The estimation of stature can also be informed by the physical characteristics of palm prints. Based on a study of 100 individuals (48 males and 52 females), Craven [27] have shown that overall length from tips to the base of the palm and the lengths from the base of the palm to the distal transverse crease are correlated to the stature of the individuals.

To provide fingerprint examiners with additional numerical support for their assessment of level 1 features, fingerprint patterns were manually classified in a set of 24,104 fingerprints [28]. In the study the relative frequencies of occurrence of 35 different fingerprint patterns have been obtained.

*Adermatoglyphia* refers to the absence of friction ridge skin on the epidermal surface of the hands and feet [29]. It can be caused by different rare syndromes (at birth) or medications such as capecitabine (brand name, Xeloda) used in the colorectal cancer treatment [30]. In an Iranian study, fingerprint changes were observed in 25 (67.6%) of the 37 patients who were treated with capecitabine and none in the comparison group without capecitabine treatment [31]. The absence of legible ridges has impact on border control (immigration and travel) and can cause undue discrimination. Specific immigration protocols should be established to deal with these cases.

Drahanský and Kanich [32] give an extensive study on the skin disease and their impact on the appearance of friction ridge skin and their usage for identification purposes.

#### 2.1.2 Level 2

Level 2 features and in particular minutiae received limited research attention compared to the previous reporting period. A few publications on palm prints brings new data to the table [33].

Dot and incipient ridges have been studied from an algorithmic perspective taking advantage of a deep convolutional neural network (CNN) named DeepDot [34]. No doubt that such algorithms can open new research avenues for a systematic acquisition of data to help forensic practitioners to assess the specificity of fingerprint features.

The field of fingerprint synthesis received a regained level of attention thanks to the use of Generative Adversarial Networks (GANs) [[35], [36], [37], [38], [39]]. It offers opportunities to create marks and prints without the need to release personal biometric data and use them to assess biometric systems and potentially forensic training purposes. It is the perfect privacy by design concept. PrintsGAN, one of the latest generators [40], is a synthetic fingerprint generator capable of generating very realistic fingerprints (512 × 512 resolution) along with multiple impressions for a given finger. GANs are also used to generate marks [41] or prints presenting level 3 features [42].

Finally, Wang et al. [43] proposed a method to compute match probabilities associated with fingerprint comparison (print to print).

#### 2.1.3 Level 3

Kaur and Dhall [44] studied the reproducibility and reliability of pore inter-distances and angles. Note though that the study was done on pristine prints obtained from 4 individuals. The applicability of these data to marks remains an open question.

At the border between detection and identification, we note the use of a Reflected Ultra Violet Imaging System (RUVIS) to systematically capture marks on non-porous surface and search them against biometric records taking advantage Convolutional Neural Networks (CNN) algorithms based on level 3 features [45]. CNN are undoubtedly the new architectures to successfully deal with the extraction and matching of level 3 (more specifically pores) features [[46], [47], [48], [49], [50], [51], [52], [53]]. Combining level 2 and level 3 features improves the latent recognition rate in comparison to the minutiae matching [54].

High quality recording of level 3 features from prints can be obtained by placing the fingers on nitrocellulose membranes, which is further processed in water [55]. These impressions are claimed to offer higher persistence than the conventional inked or livescan acquisitions.

Latent print matching algorithms received good attention as well again, taking advantage of deep-learning techniques. For example, in Ref. [47], the authors propose an end-to-end deep neural network for information quantity prediction from the fingermark. This predicted information quantity is further used to reduce the candidate list. Premk et al. [56] used CNN to segment marks from their background. Cao et al. [57,58] used convolutional autoencoders for mark enhancement, ridge flow estimation and minutiae extraction to develop an end-to-end system. The matching accuracy of this system is on par with cost-off-the-shelf systems.

Tabassi et al. [59] used CNN to detect altered fingerprints with higher accuracy compared to previous efforts (True Detection Rate above 99% for a False Detection Rate of 1%).

### 2.2 Measuring quality and suitability of fingerprint impressions

The performance of AFIS systems is heavily conditioned by the quality of the prints obtained from ten-print cards (obtained by inking or livescan). The NIST developed two generations of algorithms aimed at measuring the quality of prints, namely NFIQ1 and NFIQ2 [60]. The link between both metrics (allowing a better interoperability) has been studied by Galbally et al. [61].

The measure of the quality of marks offers the possibility of an appropriate triage of marks facing an AFIS system or in the manual comparison workflow, either to assess the suitability of the mark or to decide on the appropriate quality assurance regime. A series of algorithms are now available:

– An open-source solution developed by Oblak et al. [62,63] provides a quality aggregation method capable of fusing together multiple predicted quality values from an ensemble of quality assessment models. It includes an unconstrained CNN predictor that outperforms handcrafted features.
– A solution distributed in the US, part of the FBI's Universal Latent Workstation (ULW) called LQMetrics developed jointly between the FBI and Noblis [64]. LQMetrics is based on the training of an ML (Machine Learning) classifier against quality labels provided by fingerprint specialists from features extracted from images (local quality map, minutiae and an FFT-based implementation of LFIQ). LQMetrics delivers a quality map and a series of quality scores including a probability of a rank-1 correspondence on AFIS.
– Swofford et al. [65] proposed the DFIQI tool which measures the clarity of friction ridge features (locally), evaluates the quality of impressions (globally), and maps them to three distinct value scales (value, difficulty and complexity, as defined by Eldridge [66,67]). In an operational environment, the tool is intended to provide an empirical foundation to support experts' subjective judgments and to provide transparency to the overall quality of a given mark.

Quality measure algorithms have a wider application that is also explored in recent research. When competing detection techniques are to be compared, automatic quality measures can complement if not supplement human quality assessment [68]. The general clarity metric of the LQMetric algorithm provided the most promising score separations for automatic assignment of fingermark quality compared to other tested quality algorithms. The measurement of ridge density has also been proposed in that context, without much success yet [69].

### 2.3 Measuring examiner's performance

Eldridge aimed at understanding the value decisions that followed the analysis phase [66]. Her research allowed to show that examiners tend to be variable in their suitability determinations, and that suitability itself is a multifaceted decision. Four aspects of suitability need to be distinguished depending on the intended purpose: hence for scales named *value*, *complexity*, *AFIS suitability* and *difficulty* [70]. Taking advantage of machine learning techniques, the researchers have proposed a predicting model for value decisions based on automatically extracted quality or selectivity measures in conjunction with a limited set of user inputs [67]. The model achieved accuracy at a similar level to that of examiners asked to make the same suitability determinations across all four scales.

Following the black box studies published on fingermarks (see our previous reports), palm marks identifications have been subject to similar research [71] in which 226 latent print examiner participants returned 12,279 decisions over a dataset of 526 known ground-truth pairs. 2 false identification decisions were reported (a false positive error rate of 0.7%) for 552 false exclusion decisions (false negative error rate of 9.5%). As already identified overall and across all studies, the decision thresholds currently exhibit a preference for preventing erroneous identification errors at the expense of preventing erroneous exclusion errors. The study of anatomy of some of the errors in the above palm study has confirmed how mind-sets acquired during the analysis phase can cause wrong decisions [72]. As Spellman et al. put it [73]: “In feature comparison judgments, such as fingerprints or firearms, a main challenge is to avoid biases from extraneous knowledge or arising from the comparison method itself.”

In their investigations for the reasons for potential causes of disagreement between fingerprint experts, researchers have identified the following factors [74] in addition to the experts themselves: the quality of the images (mark and print), operating on borderline decisions using categorical conclusion scales, and the granularity of the conclusions scale. The authors have observed that the use of a three-level categorical comparison conclusion scale (ID, inconclusive, exclusion) may overstate or understate the reproducibility of conclusions, when compared to a seven-level scale. This point will be discussed in more details in the next section.

Examiners have shown robust abilities to encode marks using the color-coded annotation system known as GYRO [75]. For example, when 18 fingerprint examiners marked ridge events indicating they were “highly confident” in their existence over 300 marks, they were accurate approximately 96% of the time. These encodings were also in line with the measurements carried out using the DFIQI software [65].

Testing examiners using marks and prints offering a potential for wrong associations (colloquially named close non-match, CNM) can be seen as a sort of the worst-case scenario. In these settings, it was expected to see the false positive rate increasing. Koehler and Liu [76] have tested 135 agencies in China and obtained a false positive rate of respectively 15.9% and 28.1% respectively for two specific cases of CNMs. Li et al. [77] have searched a set of 245 simulated fingermarks (i.e., delta area of various image qualities) among a database of 6.964 million individuals. They identified that about 15% of searches led to the detection of CNMs in the first 100 candidates.

Using eye-tracking techniques Busey et al. [78] explored how wrong exclusions decisions could be explained. Using data acquired on 122 fingerprint examiners, they showed that missed identification can be explained by a shorter comparison time, fewer regions visualized on the images and fewer attempts to find potential correspondence during comparison. Hence cursory comparisons and mis-localizations are the two main explainers for missed associations. Another study addressed the eye gaze patterns of examiners (compared to novices) while they mark features and compare a latent with an exemplar fingerprint using ACE procedure [79]. Experts outperformed novices and made no false identification with a higher number of wrong exclusions.

Proficiency testing of examiners is one of the pilar of a quality management system. In the fingerprint community, the level of difficulty of the commercial tests has often been judged as too low (easy). Anecdotally, three assistant public defenders participated in the Collaborative Testing Services (CTS) Test No. 18–5161 and completed the test without committing any false positive errors [80]. They call to undertake a meaningful review of current proficiency testing practices. The timid response by Laskowski [81] attempted to draw the attention on the high false negative rate and the potential non-independence of the test takers. Blind proficiency tests have been carried out at the Houston Forensic Science Center (HFSC) [82] among them tests involving fingerprints [83].

Numerous studies aimed at understanding the cognitive and perceptual processes involved in fingerprint visual comparison tasks. They served to demonstrate (a) the benefit of perceptual training based on statistically rare features [84]; (b) the existence of a reliable visual comparison ability by experts compared to novices [85]; (c) the existence of a prevalence effect in the sense that experts and novices alike more often misjudged non-matching pairs as “matches” when non-matches were rare [86]; (d) the benefit of distributing decisions to groups of raters who independently assess the same information [87], in other words adopting a truly blind verification process; (e) the efficiency of fingerprint experts at locating specific targets on fingerprint images compared to novices, hence testifying to a domain-specific expertise [88] (f) empirical evidence that lineups (i.e. embedding the corresponding person of interest's prints among known non-source prints); promoted conservative decision-making [89].

The selection of skilled candidates to join fingerprint services is a difficult step and not much is known about the appropriate skills to measure. Ignaszak [90] provides some guidance. Individuals showing some personality traits, such as *cognitive closure* (the attitude of seeking and possessing specific knowledge in order to reduce uncertainty) and *controllability* (the ability to control internal and external influences), have shown better performance. That being said, the correlations reported are quite low.

### 2.4 The identification process and the presentation fingerprint evidence in court

The standard to be reached to decide that there is sufficient agreement between a mark and print to conclude is still a matter of debate. The situation in Poland is well described: A numerical standard between 10 and 12 matching minutiae is the minimum requirement for an identification conclusion [91]. According to the authors, the standard is rather limiting when specific features (e.g. rare combination of minutiae) are observed. They introduce the project “Mapping of friction skin ridges impressions” aiming at taking advantage of both a numerical approach (i.e. based on a numerical standard) and a holistic approach (i.e. sufficiency left to the examiner's appraisal). A dedicated software allowing to weight individual minutia is under development.

There is still a constant debate regarding the nature of conclusions reached in the context of human identification. Biedermann and colleagues clarified once again that the conclusion of identification should be interpretated as a decision that entails more than the forensic observations but incorporate an assessment of the prior probability of identity and a value assessment of the consequences of a correct or a false conclusion [92,93].

In their analysis of the Department of Justice (DOJ) Uniform Language for Testimony and Reporting (ULTR),^1^ Cole and Biedermann [94] observed that the choices of the expression of conclusion fail to observe decision theory principles and coherent probabilistic principles. They observed (p. 592) that “leaving identification decision authority to scientists would mean to let them continue to impose, implicitly, their unsubstantiated value assessments on a legal system that operates in deferential mode” and asked for the sole consideration of the weight to be assigned to the comparison findings. This view is shared by a number of academic scholars but perceived as threat by some practitioners. Scarborough put it as follows in the introduction of their book [6] (p.7): “If the shift away from confident conclusions to probabilistic statements or mere associations, it will eventually make fingerprint evidence superfluous”. The thesis by Swofford [95] is giving some forward-looking insights into the development and implementation of computational algorithms in fingermark examination. Leaving aside the development of dedicated tools (FRSTAT and DFIQI), Swofford et al. [96] analysed the challenges and fears expressed by fingerprint examiners facing the arrival of probabilistic reporting. They unsurprisingly concluded that “if probabilistic reporting is to be adopted, much work is still needed to better educate practitioners on the importance and utility of probabilistic reasoning in order to facilitate a path towards improved reporting practices”. The opinions of the stakeholders have been also teased out [97]. Swofford [95] stressed rightly that the challenges are more in the implementation that in the development of statistical models and gives eight recommendations to strengthen the foundations of friction ridge examination and improve our understanding of the reliability of evidence, to name a few: availability of the developed models to all stakeholders; a regulation by an independent authority including an oversight of the examination, interpretation and reporting practice; the adoption of minimum requirements standards for education.

An alternative scale of conclusion in the fingerprint domain with two additional values (i.e., support for different sources and support for common sources) has been proposed by the Friction Ridge Subcommittee of OSAC [98]. The document currently sits with the Academy Standard Board (ASB of the AAFS) in its third round of review.^2^ The formulation of source identification has been adapted to convey the concept of extremely strong support for one proposition (same source) versus another (different source), and two additional conclusions to qualify traditional inconclusive conclusions with degrees of support in favour of one way (same source) or another (different source). Carter et al. [99] demonstrates the utility of this expanded conclusion scale. According to them, the strengths of expanded conclusion scales outweigh the limitations, but care must be taken with their implementation. A further study contrasted fingerprint, toolmarks and footwear marks [100]. It confirmed that examiners become more risk-averse with expanded scales (both on the source identification and source exclusion) and conclusion statements that contains “extremely strong support” are reserved for comparisons with the highest amount of support. It has been shown that examiners are more risk averse than members of the general public [101].

Koehler [102] identified 20 threats to the validity of forensic source conclusions. It includes key aspects that are covered in this report such as: the absence of difficulty metrics, the individual differences between examiners, the absence of accuracy measurements and objective standards, the lack of independent verification procedure, and the risk of cognitive bias. A must read in our opinion to prepare to testify in court.

As well-argued in his commentary, Neumann [103] stresses on the need for practitioners to have some general notions of logic, inference, decision-theory and the terminology they refer to. In a survey involving 480 practitioners across 19 different countries, ACE-V is referenced in approximately 87% of laboratories (mainly fingerprint related activities) [104]. But as the authors stated (p. 275): “there appears to be significant variability in terms of the basis of the policies and procedures and application when performing comparative examinations. Consequently, although ACE-V provides a common description, there is a need to establish a standardized application within disciplines and a common basis across disciplines”.

Another aspect of fingerprint evidence that received some deserved attention is the ability to infer activities (manipulations) from the observations and positioning of the marks detected on various substrates [105]. De Ronde and colleagues investigated the specific case of marks on letters to help inform, based on the positioning of the marks, if the person who touched the surface wrote the letter or read it [106]. The method takes advantage of heat maps of detected marks and quadratic discriminant analysis to help with the classification. Out of 50 analysed letters, 48 were classified correctly, an accuracy of 96%. On knives, the researchers used a Bayesian network informed by experimental data to guide as to whether the object was used for an stabbing assault or for a legitimate bread cutting usage [107].

### 2.5 AFIS systems operations

As mentioned previously, we didn't aim at covering all the scientific literature pertaining to AFIS systems. We chose to highlight a few entries that either testify to the current research trends or have a direct impact on the forensic science operations. A short review has also been published [108].

**Trends in AFIS research** – The use of deep learning techniques using Convolutional Neural Networks (CNNs) is spreading on numerous areas of fingerprint recognition. We note its increased performance for the classification of general patterns according to the Galton-Henry main classes [109] with an accuracy of about 83% using a pre-trained Googlenet. Other researchers [110] have used CNNs for predicting finger number or the sex of the donor with good performance. Finally, AFIS systems are also benefiting from the advances in deep learning techniques [111].

**AFIS operations** - Under the auspices of the Joint Research Centre, a report assesses the technology readiness and availability of new functionalities (fingermarks and palm marks) for their integration into the Schengen Information System (SIS) [112].

Dealing with infant fingerprint images is challenging for current AFIS systems. Images of prints obtained from infants can be specifically enhanced [113]. Dedicated acquisition and matching systems have been designed to deal with infant prints [114].

**Overlapping impressions** – dedicated algorithms are proposed to handle situation where marks are superimposed [115].

**Livescan sensors** – Kumari et al. [116] have shown the degrading effects of alcohol-based hand sanitizer, greasy lotion, and viscous oil on touched livescan devices. A touchless system would mitigate these issues. We note the development of large field of view quadratic metalenses allowing the acquisition of 5 mm fingerprint with features of the order of 100 μm at a 2.5 mm distance [117] and the search for algorithm allowing the capture of marks at a distance with mobile devices [118]. The application of NFIQ2 as a quality metric for touchless system has been shown [119]. Under constrained capture conditions NFIQ2 is found to be an effective tool for touchless fingerprint quality estimation if an adequate preprocessing is applied. We will come back later to the topic of identification of individual from friction ridge skin images gathered from social media, but it is worth mentioning here that biometric systems are developed to handle verification using selfies taken from fingers [120,121].

In their search for a cost-effective inkless method to replace the conventional black ink method, De Alcaraz-Fossoul and Li [122] proposed a method using a commercially available alcohol-based hand sanitizer gel as the medium and standard thermal paper as the substrate.

Finally, we draw the attention to the excellent review paper on subcutaneous biometric techniques [123]. Capturing papillary information at the dermis level using optical coherence tomography (OCT) offers new ways to mitigate the risk of transactions with forged fingers or the use of surface-altered fingerprint to avoid detection. OCT also improves the detection of pores.

### 2.6 Fake fingerprints, presentation attacks and security

Presentation attacks taking advantage of spoof fingers is still considered as a major treat against biometric systems and for document identification (typically in Asian countries). Kumar and Pryanka offer an overview of live detection techniques to secure fingerprint recognition system from spoofing attacks [124]. The section on fingerprint biometrics from Marcel et al. [125] gives also a very detailed state-of-the-art.

From 2009, the Fingerprint Liveness Detection Competition (LivDet) aims at assessing the performance of the state-of-the-art algorithms according to a rigorous experimental protocol. The 2017 results show a steady increase in spoof detection performance (up to 95% accuracy) [126,127]. The accuracy measured during the LivDet test of 2021 reached 99%. It is often assumed that spoofs obtained with the cooperation of the donor present higher threat than spoofs produced without the cooperation of the donor (e.g., spoofs prepared from latent marks). Recent tests have shown that the threat is of the same level [128].

It will come as no surprise that CNNs have shown excellent performances to distinguish genuine prints from forged ones (see for example [[129], [130], [131], [132], [133]]), but with a generalization capability (application to other types of forgeries) that is very dependent on the training samples used (see Ref. [134]). Indeed “one of the major limitations of current spoof detection methods is their poor generalization performance across “unknown” or novel spoof materials, that were not used during training of the spoof detector.” [135]. Grosz, Chugh and Jain [135,136] resort to augment the CNN spoof detector training samples with synthetic spoof images covering various materials and backgrounds. More traditional approaches (with ensemble techniques) showed good performance as well [[137], [138], [139]].

It is a never-ending fight against the arrival of new material that can be used to prepare spoof fingers. New types of spoofs are one of the major threats faced by latest generation, deep leaning based, presentation attack algorithms. Saguy et al. [140] presented how pro-active forensic science allows to create innovative and performing materials mimicking the attributes of friction ridge skin. The novel materials prepared were hydrogels based on polyethylene glycols (PEGs) that were chain-extended. They showed good performance in deceiving security systems (including systems equipped with presentation attack detection), considerably higher than that of spoofs produced from commercial materials.

Some authors are suggesting tests linked with the fingerprint residue. C stain method successfully developed forged fingerprints on both porous and non-porous surfaces, whereas it failed to develop real fingerprints on any surface [141]. The C stain and Hertzberg stain allowed to detect marks left by forged fortified PVA-based fingerprints [142].

Some early research explored the possibility of detecting forged marks (as opposed to forged prints) [143]. CNN approaches generally showed reduced error rates compared to hand-crafted features.

## 3 Fingermark composition

During the covered period, 55 papers were published in relation to fingermark composition, which were dispatched as follows: fingermark composition and aging (26 pap.), donor profiling (9 pap.), and contaminations (20 pap.). The publications are equally distributed between forensic and non-forensic journals, with 27 and 28 papers, respectively. The three most popular journals were Journal of Forensic Sciences (12 pap. – 22%), Forensic Chemistry (6 pap. – 11%) and Analyst (6 pap. – 11%). Given that the analysis of fingermark composition relies on high-end analytical methods, it is expected that such publications may be of interest for other scientific fields.

One limitation of this research field is that most of the studies are conducted on limited sample sets (e.g., one donor, few depositions) or enriched secretions (e.g., sebum-rich fingermarks, excessive spiking). They must hence be considered as preliminary/pilot, and the observed trends or expressed conclusions taken with caution. Only few papers considered natural fingermarks left on realistic substrates or exposed to casework-like conditions. This is certainly an aspect of the field that could be improved, to strengthen the conclusions and improve the transposition to the practical field. In this context, the IFRG guidelines [144] were cited by five papers, only. Although these guidelines were primarily intended for people working in the detection field, the provided good practices could be helpful for the people aiming at studying the composition of fingermarks (e.g., donors, types of secretions, substrates).

### 3.1 Fingermark composition and aging

The study of fingermark composition serves two main purposes: (1) getting a better understanding of the fingermark chemistry (e.g., spatial distribution of compounds, impact of detrimental conditions) so that detection techniques could be improved, and (2) monitoring the aging of fingermarks or identifying aging markers, so that fingermarks could be dated. In this context, research has confirmed that the amount of secretion decreases with age [145].

**Fingermark composition** – Brief overview of the studies aiming at analysing the composition of fingermarks: optimization of the extraction protocol allowing the analysis of fatty acids via GC-FID [146]; investigation of the composition of fingermarks and the distribution of the molecular fractions on flat, non-porous substrates using LIFS [147]; observation of shed skin cells in fingermarks deposited on flat, non-porous substrates using dark-field microscopy [148]; description of a workflow aiming at collecting DNA and proteins from fingermarks left on glass, using an anionic surfactant [149].

The following two papers further addressed the question of fingermark composition, using natural fingermarks:

- the presence of endogenous and exogenous inorganic ions in natural fingermarks was imaged and discussed, for example with regards to the use of cosmetics [150]. The authors also confirmed the detrimental impact of water immersion on components of eccrine origin, as opposed to sebum.
- natural, eccrine- and sebum-rich fingermarks provided by more than 450 donors were analysed by UPLC-MS and GC-MS, and the gathered data processed by targeted metabolomics and untargeted profiling [151]. The authors showed that (1) the content in amino acids range from 100 ng to 10 μg per fingermark, natural ones containing 5.4 times more amino acids than eccrine-rich ones, (2) the content in lipids range from 100 ng to 100 μg per fingermark, sebum-rich ones containing 5.9 times more lipids than natural ones. Donor profiling was also carried out (See Section 3.2).

**Fingermark aging** – Brief overview of the studies aiming at monitoring the evolution with time of the fingermark composition, to identify aging markers: use of chemical assays based on amino acids [152]; demonstration that the fingermark quality cannot be used as an aging marker when fingermarks are exposed to heat for a prolonged period of time (i.e., light bulbs powered for up to 672 h) [153]; evolution of ridge width [154] and colour contrast metric [155] from powder-dusted fingermarks; use of μ-FTIR combined with chemometric tools [156]; evolution of triglycerides (triacylglycerols) with time and better comprehension of the ozonolysis-driven degradation pathway [157,158]; degradation of squalene and cholesterol upon exposure to temperatures ranging from −20°C to 100°C [159]; consideration of amino acid racemization, more particularly the enantiomeric ratio of D/l-serine, as aging marker [160]; evolution of fatty acids over a 30-day-aging period and consequences on detection techniques (i.e., powder dusting, iodine and silver nitrate) [161]. It should be noted that most of these above-cited studies considered sebum-rich fingermarks.

The following three papers further addressed the question of fingermark aging, using natural fingermarks provided by more than one donor:

- Boseley et al. showed that [162]: (1) the first molecular modifications occur quickly after the deposition (e.g., degradation and redistribution of lipids, loss of water within the first 8 h), (2) the aging mechanisms appear to be driven by unpredictable rates, challenging the possibility to use the chemistry of fingermarks to estimate the age of a fingermark without knowing its initial composition, and (3) the loss of water within the first hours ranged between 14 and 20 μg.
- considering natural and enriched fingermarks left by 150 individuals and exposed to different conditions for up to 85 days, Czech et al. visually observed that females left fingermarks with larger ridges compared to males and confirmed that the passing of time reduces the ridge width and the quantity of material available [163].
- the degradation of squalene with time was monitored using aluminium as substrate and different storage conditions (i.e., dark/light, immersion) [164]. The authors identified oxidation by-products of squalene and cholesterol, which were relatively stable over time and could hence constitute good candidates for fingermark detection techniques applied to aged fingermarks.

**Other** – Cook et al. illustrated the impact of adermatoglyphia (i.e., lack of ridge detail, sometimes combined with the absence of eccrine secretions) on the detection of latent fingermarks by common detection techniques (i.e., CA, NIN, and PD) [165]. In a similar context, Finigan et al. investigated the impact of HED (i.e., partial or complete lack of eccrine glands) [166]. In this study, 22 HED-positive and 22 HED-negative (= control) participants were asked to leave natural fingermarks on three substrates (i.e., paper, ceramic tile, and plastic). The fingermarks were then aged for six months before being processed with either [CA ➭ R6G], black magnetic powder or IND/Zn. Overall, fingermarks from HED-positive donors were of lower quality. Sun et al. confirmed the presence of biological substances in seals made of inked fingermarks, to distinguish them from forgery attempts involving the use of stamps [167].

**Relevant reviews** – Thorough review about the chemical composition of fingermarks, the impact of aging, and their detection through common detection techniques or chemical imaging [168]; extensive review covering ten years of publications related to the composition of fingermarks and emphasizing the role of MS-based techniques [169]; review about age determination techniques, based on physical characteristics and chemical composition [170].

Acronyms used: **CA** (cyanoacrylate fuming), **FID** (flame ionization detection), **FTIR** (Fourier transform infrared spectroscopy), **GC** (gas chromatography), **HED** (hypohidrotic ectodermal dysplasia), **IND/Zn** (1,2-indanedione combined with zinc chloride), **LIFS** (laser-induced fluorescence spectroscopy), **MS** (mass spectrometry), **NIN** (ninhydrin), **PD** (physical developer), **R6G** (rhodamine 6G), **UPLC** (ultra-performance liquid chromatography)

### 3.2 Donor profiling

Donor profiling is the ability to provide information about individuals from the analysis of their fingermarks (e.g., sex, diet, habits, ethnical origin). The underlying objective is to provide intelligence to investigators, in combination to ridge details and touch DNA.

**Sex determination** – Overview of the approaches aiming at determining the sex of donors from their fingermarks:

- analysis of the peptide and protein content of natural fingermarks by MALDI-MSP [171], identification of limitation factors (e.g., presence of PEG-based polymers from cosmetics) and claimed success rates ranging from ∼70% to 85%, according to the chosen scoring strategy
- solubilization of fingermarks and introduction into a microfluidic platform containing a colorimetric test targeting arginine [172] – claimed success rate above 90%
- solubilization of fingermarks and measure of the fluorescence intensity induced by IND [173] – unsuccessful
- analysis of sebum-rich fingermarks on glass by Raman spectroscopy, combined with chemometrics [174] – claimed success rate above 80%.

**Other traits** – Overview of the approaches aiming at determining other traits associated to the donors of the fingermarks:

- analysis of the peptide and protein content of natural fingermarks by MALDI-MSP to determine the age of the donor [171] – unsuccessful
- identification of haemoglobin variants in bloody fingermarks, using MALDI-MSP, to provide information about the donor blood profiling [175] – See Section 4.5.1
- monitoring of the relative amounts of saturated and unsaturated triacylglycerols as markers of health (i.e., diabetes), diet, and exercise [176] – mitigated results
- microbial/bacterial profiling as indicator of various traits (e.g., sex, age, ethnicity, home location, diet, use of cosmetics) [177] – mitigated results
- analysis of natural, eccrine- and sebum-rich fingermarks provided by more than 450 donors, using UPLC-MS, GC-MS, targeted metabolomics, and untargeted profiling [151]. The authors showed that (1) the metabolites involved in the GMP degradation pathway could help predicting the sex (claimed success rate of ∼78%), (2) nicotine and cotinine could help predicting smoking habits (claimed success rate above 90%).

**Relevant review** – Personal opinion about the chemical profiling of fingermarks, especially using MALDI-MS [178].

Acronyms used: **GC** (gas chromatography), **GMP** (guanosine monophosphate), **IND** (1,2-indanedione) **MALDI** (matrix-assisted laser desorption ionization), **MS/P** (mass spectrometry/profiling mode), **PEG** (polyethylene glycol), **UPLC** (ultra-performance liquid chromatography)

### 3.3 Contaminations

The aim of this report was not to cover extensively the presence of exogenous compounds in fingermarks, encompassing fields such as medicine or road safety (e.g., drug screening cartridges). Consequently, only the studies addressing the detection of exogenous compounds found in fingermarks left on substrates were reported.

**Drugs and explosives** – Brief overview of the studies aiming at detecting the presence of drugs or explosive traces in fingermarks: imaging of drug- and explosive-contaminated fingermarks using LADI-MSI [179]; imaging of drug- and clay-contaminated fingermarks using a gold-coated nanostructured silicon substrate compatible with SALDI-MSI and SERS [180,181]; imaging of explosive-contaminated fingermarks using DART-HRMS and MALDI-MSI [182]; detection of drugs and lead in contaminated fingermarks using Raman spectroscopy [183] and SALDI-MS [184]; detection of explosives in contaminated fingermarks using O-PTIR spectromicroscopy [185]; detection of hypolipidemic drugs in fingermarks spiked post-deposition, using UPLC-Q-TRAP-MS [186]; detection of caffeine, lead, lead oxide, and titanium dioxide in fingermarks using XPS [187]; detection of cutting agents in drug-contaminated fingermarks using HPLC-ESI-qToF-MS [188]; detection of amphetamine in contaminated fingermarks lifted by adhesives and analysed by ToF-SIMS [189]; detection of metoprolol and its metabolite in contaminated fingermarks using LC-MS/MS [190].

The following three papers addressed the question of drug- and explosive-contaminated fingermarks, considering casework-related fingermarks or natural secretions:

- in the context of smuggled synthetic-cannabinoid-soaked papers in prisons, Caterino et al. showed that it was still possible to identify the presence of drugs on the items after processing them with DFO and NIN, both applied by spray [191]. In the same context, Gulecki et al. showed that the application of amino acid reagents by immersion led to considerable loss of drug molecules from the processed items [192].
- to distinguish the contact with cocaine from its consumption, Costa et al. applied various analytical techniques presenting imaging capabilities (i.e., DESI-MSI, MALDI-MSI, ToF-SIMS) to contaminated fingermarks [193]. The authors showed that all three techniques were able to distinguish the two scenarios, mostly due to the differences in the spatial distribution of cocaine with regards to its metabolite.

**Condom lubricants** – The detection of condom lubricant traces in fingermarks, combined with the possibility to determine the brand of the condom, has been addressed by van Helmond et al. [194]. Using DESI-MSI, the authors demonstrated that molecular compounds linked to condom lubricants (e.g., PDMS, PEG, octoxynol-9 and nonoxynol-9) could be detected and imaged in CA-processed fingermarks. Considering a database of 32 different condoms, their classification model reached an accuracy of ∼90% when applied on lubricant-contaminated fingermarks.

**Household products** – Brief overview of the studies that considered contaminations with household products: use of carbon-based black powder [195] or titanium dioxide-based white powder [196] as matrixes to image fingermarks contaminated with various household products (e.g., drug, bug spray, sunscreen, wine, fruits) by HRMS MALDI-MSI; airflow-assisted DESI-MSI to image fingermarks contaminated with inkpad or sunscreen [197].

**Metal traces** – The transfer and persistence of metals in fingermarks has been investigated by Boseley et al., using XFM [198]. Considering different scenarios, the authors showed that the handling of the following items led to an increase of metal traces along the fingermark ridges: (1) for a gun barrel: iron and lead – heterogeneously distributed, (2) for a brass cartridge case: copper, and zinc – homogeneously distributed, (3) for a party sparkler: iron, cobalt, nickel, bromine, and barium. Considering this last scenario, the authors showed that washing the hands resulted in a significant loss of the transferred metal traces.

Acronyms used: **CA** (cyanoacrylate fuming), **DART** (direct analysis in real time), **DESI** (desorption electrospray ionization), **DFO** (1,8-diazafluoren-9-one), **ESI** (electrospray ionization), **HPLC** (high-performance liquid chromatography), **HRMS** (high-resolution mass spectrometer), **LADI** (laser ablation direct analysis in real time imaging), **LC** (liquid chromatography), **MALDI** (matrix-assisted laser desorption ionization), **MS/I** (mass spectrometry/imaging), **NIN** (ninhydrin), **O-PTIR** (optical-photothermal infrared), **PDMS** (polydimethylsiloxane), **PEG** (polyethylene glycol), **qToF** (quadrupole time of flight), **Q-TRAP** (triple quadrupole/linear ion trap), **SALDI** (surface-assisted laser desorption ionization), **SERS** (surface-enhanced Raman spectroscopy), **SIMS** (secondary ion mass spectrometry), **ToF** (time of flight), **UPLC** (ultra-performance liquid chromatography), **XFM** (X-ray fluorescence microscopy), **XPS** (X-ray photoelectron spectroscopy)

## 4 Fingermark visualisation

### 4.1 Research interests overview

**Fig. 1:**
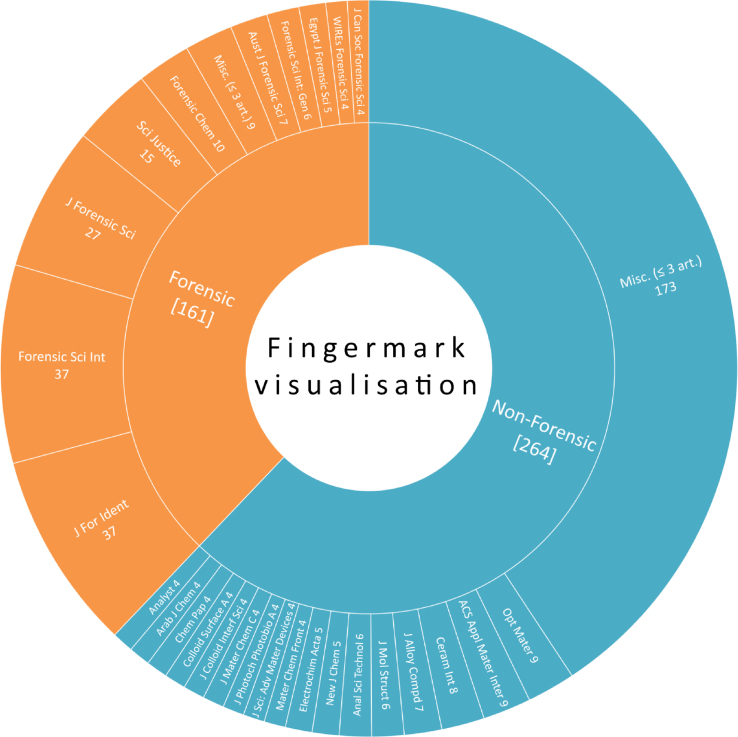
Sunburst representation depicting the publication portfolio associated to the 425 papers related to fingermark visualisation. The figures represent the number of papers associated to each journal. The sub-category “Misc. (≤3 art.)” regroups all the papers that have been published in journals that have published three papers (or less) during the covered period.

During the period covered by this report, 425 papers dealing with fingermark visualisation were collected, representing an increase of 16% compared to the 2016–2019 report [1]. Fig. 1 illustrates the repartition of those papers among scientific journals, classified as “forensic” and “non-forensic” according to their editorial scopes and target readerships. It clearly appears that most of the papers were published in non-forensic journals (264 pap. – 62%), as compared to forensic journals (161 pap. – 38%). This trend was observed for the first time in the 2016–2019 report, and it raises again questions about the targeted readership or the loss of forensic considerations in favour of a technological drift far from casework constraints. Similar to the previous report, the three most popular forensic journals are Forensic Science International (FSI – 37 pap.), Journal of Forensic Identification (JFI – 37 pap.), and Journal of Forensic Sciences (JFS – 27 pap.). FSI, which was leading the field for a long time, is now sharing the top step of the podium with JFI. Overall, these three journals represent 24% of all the papers that were published in relation to fingermark visualisation, but they encompass more than 60% of those published in forensic journals. This confirms that these journals represent leading choices to target the forensic community. Conversely, most of the papers associated with non-forensic journals (mostly chemistry-oriented ones) were found in journals having published less than three papers related to fingermark visualisation during the covered period (i.e., 173 papers published in ∼130 different journals). The three most popular chemistry-oriented journals were Optical Materials (9 pap. – 2%), ACS Applied Materials & Interfaces (9 pap. – 2%) and Ceramics International (8 pap. – 2%). Given that these three differ from the ones identified in the previous two Interpol reports, this confirms that no chemistry-oriented journal can currently be considered as a leader in the field of fingermark visualisation.

**Fig. 2:**
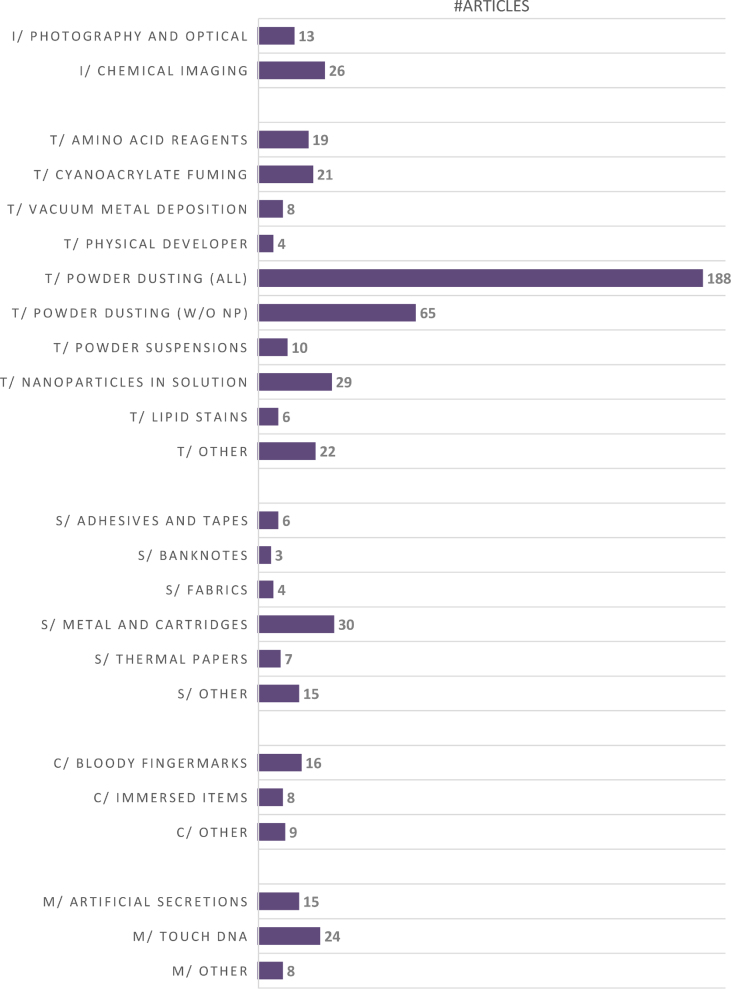
Subtopics (and associated number of papers) covered in the five main sections of this report (i.e., photography and chemical imaging – I, detection techniques – T, substrates – S, contextual situations – C, and miscellaneous – M). Abbreviation used: w/o NP (without nanoparticles).

**Fig. 3:**
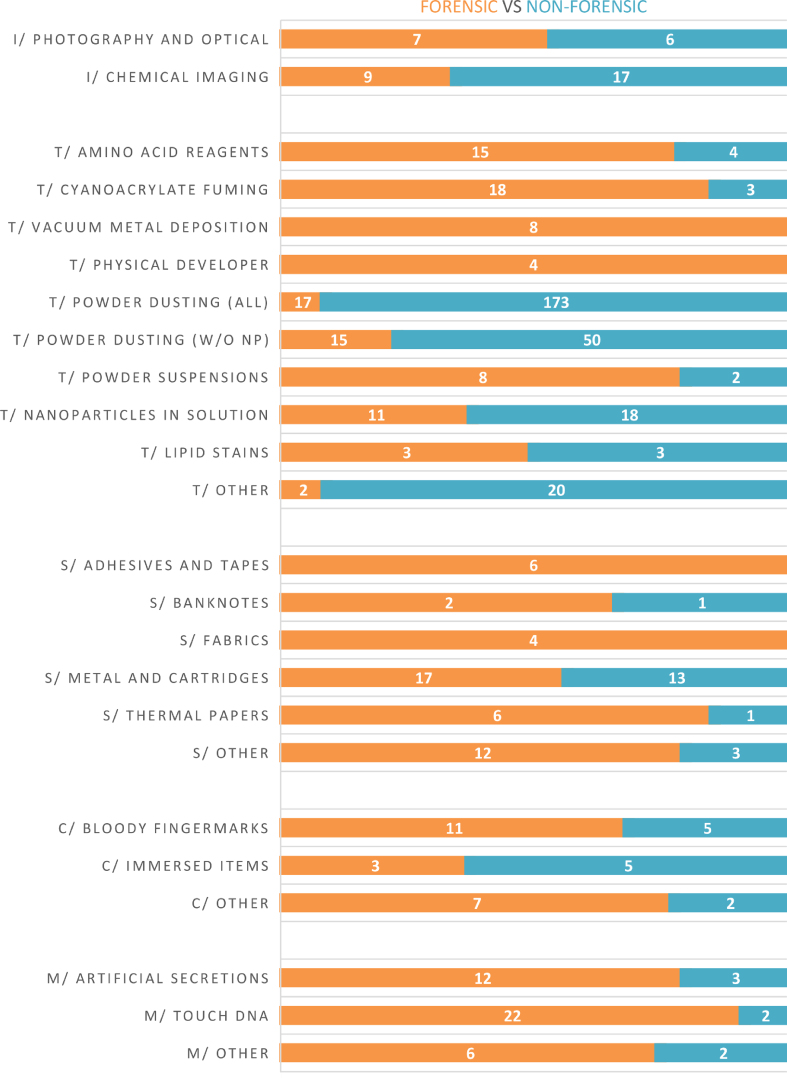
Proportions of articles published in forensic (orange) and non-forensic (blue) journals, for each of the subtopics covered in this report. Abbreviation used: w/o NP (without nanoparticles).

With regards to the covered topics, the 425 papers have been dispatched over five main sections: Photography and chemical imaging (I) – Section 4.2, Detection techniques (T) – Section 4.3, Substrates (S) – Section 4.4, Contextual situations I – Section 4.5, and Miscellaneous (M) – Section 4.6. Fig. 2 provides an overview of the number of papers published in each of these sections. Fig. 3 depicts the distribution between forensic and non-forensic journals for each subtopic. In these two Figures, the sum of all the reported numbers exceeds 425 because some papers were distributed into more than one subtopic.

As it can be seen in Fig. 2, the three main topics of interest for the last three years have been “powder dusting” (188 pap. – 44%), followed by “metal and cartridges” (30 pap.) and “nanoparticles in solution” (29 pap.). The huge gap between powder dusting and the following topics has already been emphasized in the last report. We can only express concerns with regards to this trend because most of the papers dealing with powdering recommend the dusting of nanoparticles (123 pap.) or show no consideration for forensic constraints or actual needs (e.g., freshly deposited sebum-rich marks). In the previous report, we already emphasized that one third of the papers published in fingermark visualisation recommended the dusting of nanoparticles. This practice goes against all ethical considerations for practitioners in terms of health and safety issues, and against any scientific strategy dedicated to fingermark detection. Given that the trend has not reversed, we have decided not to cite any of the 123 papers recommending the powdering of nanoparticles in this report. At the exception of three papers, they were all published in chemistry-oriented journals. When removing the dusting of nanoparticles from the powder dusting category, the number of papers drops to 65 (Fig. 2). Among the historical topics of interest, we can emphasize the success of fingermark recovery on metal and cartridges (30 pap.), cyanoacrylate fuming (21 pap.), amino acid reagents (19 pap.), and bloody fingermarks (16 pap.). Contrarily to the previous report, no paper addressed the question of fingermark recovery on skin/leather or in the context of arson. Fig. 3 provides an overview of the publication trend observed for each subtopic, with regards to forensic and non-forensic journals.

**Good practices** – When writing this report, the perceived feeling was that more researchers considered the use of natural secretions, as opposed to enriched or spiked ones. Also, the International Fingerprint Research Group – IFRG guidelines [144] were cited by 90 papers, representing 21% of the papers dealing with fingermark visualisation. Several authors identified their study as “Phase 1” (i.e., preliminary/pilot) or discussed their methodological choices through them. The percentage cited above is twice the one observed in the 2016–2019 report. A positive way of looking at these figures is that the guidelines become more popular, which benefits the community and the research quality. A more critical way at looking at these figures is that several authors cite these guidelines to say that they diverged from them: for ease, to ensure good results, etc. Nevertheless, the use of natural fingermarks and the awareness of the IFRG guidelines contribute to strengthen the research in the field, and it is expected the trend will continue.

**Relevant reviews** – About 30 reviews were published, but not all were cited. Only those that were identified as relevant and meaningful for readers were reported. Besides the specific reviews, we can recommend the following ones, for they addressed broad topics: summary of the research projects presented at the 2015 and 2017 IFRG meetings [199]; meta-analysis of the Interpol reports published between 2004 and 2019 [200]; evolution of the publication trends in friction ridge discipline between 2005-2009 and 2010–2014 [2].

### 4.2 Photography and chemical imaging

#### 4.2.1 Photography and optical methods

**Photography – Preliminary/Pilot studies** – Various approaches were proposed to enhance the recording of fingermarks: geometric compensation to record fingermarks left on curved surfaces [201]; ±2 EV HDR imaging to enhance the contrast of black powder-processed fingermarks left on illustrated non-porous substrates [202]; lens barrel equipped with LEDs to improve the recording of fingermarks on flat, non-porous substrates [203]; performances of DSLR compared to smartphone cameras [204]; intentional overexposure up to +3 EV to improve the visualisation of wet and blood marks on dark-coloured fabrics [205].

**Optical methods – Preliminary/Pilot studies** – A couple of optical methods were proposed to detect latent fingermarks: UV-induced luminescence HSI combined with chemometric tools to separate overlapped fingermarks left on TLC plates [206]; reflected UV polarization imaging to detect fingermarks on flat, non-porous substrates [207]; short-wave 266 nm UV laser to induce long-wave UV luminescence of fingermarks on porous substrates [208,209] – note: the authors observed some laser-induced degradation of the fingermarks and the substrates; reflected MWIR imaging to detect fingermarks on the adhesive-side of tapes, and on flat, non-porous substrates [210]; OCT to separate “overlapped” or hidden fingermarks [211] – note: the so-called “overlapped” fingermarks were actually left on four stacked glass slides.

**Mirrors –** To ease the recording of fingermarks on mirrors, Accioly proposed to remove the backing layers so that the mirror becomes transparent and behaves like a conventional glass [212]. The protective layer is removed first, using methylene chloride-containing paint remover, followed by the removal of the silver layer using 65% aqueous nitric acid.

**Observation filter** – In a very short study, Lee et al. illustrated the advantages of using interference filters compared to absorption ones [213]. In that context, the ILPF550 filter (Altlight, Korea) seemed particularly well adapted to the recording of fingermarks processed with IND/Zn and [CA ➭ BY40].

**Relevant reviews** – Use of smartphones to record fingermarks, in terms of image quality and admissibility in court [214].

Acronyms used: **BY40** (Basic yellow 40), **CA** (cyanoacrylate fuming), **DSLR** (digital single-lens reflex), **EV** (exposure value), **HDR** (high-dynamic range), **HSI** (hyperspectral imaging), **IND/Zn** (1,2-indanedione combined with zinc chloride), **LED** (light emitting diode), **MWIR** (medium wavelength infrared), **OCT** (optical coherence tomography), **TLC** (thin-layer chromatography), **UV** (ultraviolet)

#### 4.2.2 Chemical imaging

Most of the papers related to chemical imaging were preliminary/pilot studies based on limited sets of sebum-rich or contaminated fingermarks left on glass or other non-porous substrates. Consequently, an overestimation of the reported performances is expected.

**Preliminary/Pilot studies** – Airflow-assisted DESI-MSI to image sebum-rich and contaminated fingermarks on paper [197]; LADI-MSI to image sebum-rich or contaminated fingermarks [179]; MALDI-FT-ICR-MSI to image fingermarks previously covered with blood [215]; use of carbon-based black powder [195] or titanium dioxide-based white powder [196] as matrixes to image sebum-rich or contaminated fingermarks by HRMS-MALDI-MSI; combined use of DART-HRMS and MALDI-MSI to image contaminated fingermarks [182]; Raman imaging to visualize fingermarks detected by lysozyme-specific aptamer-coated gold nanoparticles [216]; Sagnac UV FT imaging spectroscopy to image fingermarks spiked with vitamins C and B6 [217]; use of a gold-coated nanostructured silicon substrate compatible with SALDI-MSI and SERS [180,181]; SECM imaging of fingermarks previously transferred to a nitrocellulose membrane [218,219]; SERS imaging of fingermarks previously detected by SMD II [220]; ToF-SIMS to flatten fingermarks left on cartridge cases [221]; ToF-SIMS combined with a neural network to separate overlapped fingermarks [222]; XPS to image the presence of lead, lead oxide, titanium dioxide, and caffeine in fingermarks [187].

**Other sections** – The following papers are detailed in other sections of this report: XFM to study the distribution of inorganic ions in fingermarks [150] and synchrotron-sourced ATR-FTIR to investigate the aging of natural fingermarks [162] – See Section 3.1; DESI-MSI, MALDI-MSI, and ToF-SIMS to distinguish contact with drugs from drug consumption [193] – See Section 3.3; DESI-MSI to classify condom lubricants in CA-processed fingermarks [194] – See Section 3.3; MALDI-MSI applied in sequence with conventional techniques to detect fingermarks on the adhesive-side of tapes [223] – See Section 4.4.1; ToF-SIMS to image fingermarks on worn RMB cotton-based banknotes [224] – See Section 4.4.2; MALDI-MS to provide intelligence about the human origin of blood [225] and haemoglobin variants [175] – See Section 4.5.1; design of a control test for MSI-based techniques [226] – See Section 4.6.1.

**Relevant reviews** – MS-based methods applied to fingermark imaging and profiling [227].

Acronyms used: **ATR** (attenuated total reflection), **CA** (cyanoacrylate fuming), **DART** (direct analysis in real time), **DESI** (desorption electrospray ionization), **FT** (Fourier transform), **FTIR** (Fourier transform infrared spectroscopy), **HRMS** (high-resolution mass spectrometer), **ICR** (ion cyclotron resonance), **LADI** (laser ablation direct analysis in real time imaging), **MALDI** (matrix-assisted laser desorption ionization), **MS/I** (mass spectrometry/imaging), **RMB** (renminbi; China), **SALDI** (surface-assisted laser desorption ionization), **SECM** (scanning electrochemical microscopy), **SERS** (surface-enhanced Raman spectroscopy), **SIMS** (secondary ion mass spectroscopy), **SMD II** (single metal deposition II), **ToF** (time of flight), **UV** (ultraviolet), **XFM** (X-ray fluorescence microscopy), **XPS** (X-ray photoelectron spectroscopy)

### 4.3 Detection techniques

#### 4.3.1 Amino acid reagents

**Preliminary/Pilot studies** – New amino acid reagents were proposed to detect fingermarks on porous substrates: alizarin and purpurin [228]; 2-substituted-1,4-naphthoquinones and 2-substituted-1,4-anthraquinones [229]; fluorescent l-tyrosine derivatives [230]. The addition of liquid glue to a NIN solution (0.02–0.05 g/ml) was proposed to prevent the darkening of thermal papers while allowing NIN to react with fingermarks [231]. The use of PVA/NIN blend nanomembranes was proposed to detect fingermarks on non-porous substrates, through a two-step approach: transfer on the membrane, followed by NIN processing [232]. Note: most of these preliminary/pilot studies relying on limited sample sets (e.g., one donor, few depositions, fresh fingermarks), an overestimation of the reported performances is expected.

**Comparative studies** – The following studies investigated the relative performance of IND in different contexts: solvent-less application of IND through the use of a vacuum oven [233]; comparison between DFO and IND/Zn in a tropical environment [234]; comparison between IND/Zn and NIN to detect fingermarks on brown paper bags and cardboard [235]; assessment of Solstice-PF as an alternative carrier solvent for IND/Zn [236]. These studies are briefly described here-below, and the most relevant observations/conclusions cited:

- the application of IND in a vacuum oven (i.e., 1 mbar and 50°C, for 5 min) was compared to the conventional process (i.e., immersion in IND/Zn followed by heating at 155–160°C for 10 s using a heat press) and the dry-transfer (i.e., item placed under two contact transfer sheets that were pre-soaked in IND/Zn and dried) [233]. The authors considered depletion series of up to one-year-old natural fingermarks left on porous substrates (i.e., office paper, cardboard, glossy magazine, and thermal paper). Non-porous substrates were also considered for vacuum IND only (i.e., ceramic tile, glass, aluminium, PE film, and AUD polymer banknotes). The main conclusions were: (1) overall, conventional IND/Zn outperforms the dry-transfer and vacuum IND; (2) similarly, the dry-transfer outperforms vacuum IND; (3) vacuum IND was able to detect some of the 1-h-old marks left on ceramic tiles and aluminium foil; (4) glossy magazine proved to be the most challenging substrate (more than 43% of undetected marks) and office paper, the least challenging one (less than 7% of undetected marks). Further research is expected to investigate the ability of vacuum IND to detect fingermarks on non-porous substrates.
- considering depletion series of natural fingermarks left on seven substrates (i.e., office paper, glossy and paper magazines, white and brown envelopes, cardboard, and joss paper), aged up to three months, and stored indoor or outdoor, Loh et al. showed that IND/Zn outperformed DFO [234]. Fingermarks left outdoor were protected from direct exposure to rain but exposed to tropical climate, which led to poor detection performances. Glossy magazine, brown envelopes, and joss paper proved to be the most challenging substrates.
- considering depletion series of natural and eccrine fingermarks left on five substrates (i.e., brown paper bags, standard cardboard, single- and double-wall cardboard, and white paper-like cardboard) and aged up to one week, Aguayo and Pohl showed that IND/Zn outperformed NIN in all cases [235]. Single- and double-wall cardboards proved to be challenging substrates, with both reagents leading to poor performances.
- considering depletion series of natural fingermarks left on three substrates (i.e., copy paper, brown paper, and newspaper) and aged up to one month, Zhao et al. compared four HFE7100-based IND/Zn formulations (i.e., CAST 2011 and 2014, UNIL, and UTS) as well as one Solstice-PF-based formulation. The main conclusions were: (1) the CAST2014 formulation was assessed as being the most performant HFE7100-based formulation, in terms of ridge clarity and fluorescence intensity, followed by the UNIL one; (2) the use of Solstice-PF as carrier solvent for the CAST2014 formulation allowed to detect +2% fingermarks on copy paper, and +10% on brown paper and newspaper, compared to HFE7100. Further studies are expected to confirm these trends.

**Old documents** – The timescale for fingermark detection with amino acid reagents has been significantly increased, for 80-year-old documents were successfully processed with IND/Zn [237]. In this study, people in Australia and UK were asked to process several documents (e.g., correspondence, invoices, Christmas cards, notebooks, envelopes) which were categorized by their age (i.e., contemporary, 35–40 years, and 75–90 years). Despite the decrease of success rates over time, handling evidence and *identifiable* fingermarks were detected on several documents. Amino acid diffusion was also observed on some papers (e.g., postcards and pre-printed notebooks), hypothetically due to cyclic temperature and humidity conditions. Also, the success rate was higher for the documents processed in Australia compared to UK, which could emphasize the role played by climatic conditions in fingermark preservation.

**Miscellaneous** – Computational chemistry was proposed to help designing new amino acid reagents presenting enhanced optical properties [238].

**Other sections** – The following papers are detailed in other sections of this report: use of IND/Zn to emphasize grabbing areas on fabrics [239] – See Section 4.4.3; performance of amino acids reagents to detect fingermarks on different kinds of gloves [240] – See Section 4.4.6; impact of ethanol on the quality and recovery of fingermarks by amino acid reagents and ORO [241] – See Section 4.5.3; design and assessment of positive control tests for amino acid reagents [[242], [243], [244]] – See Section 4.6.1.

Acronyms used: **AUD** (Australian dollar), **CAST** (Centre for Applied Science and Technology, UK), **DFO** (1,8-diazafluoren-9-one), **IND/Zn** (1,2-indanedione/combined with zinc chloride), **NIN** (ninhydrin), **ORO** (oil red O), **PE** (polyethylene), **PVA** (polyvinyl alcohol), **UNIL** (University of Lausanne, CH), **UTS** (University of Technology Sydney, AU)

#### 4.3.2 Cyanoacrylate fuming (CA)

**Preliminary/Pilot studies** – A tetraphenylethene-based molecule was proposed as a new CA dye [245]. DMAB 0.5% (w/w) [246], salicylic acid, and anthranilic acid [247] were identified as promising fluorophores to be integrated in a one-step CA process. The detection performances and DNA recovery abilities of four CA products (i.e., traditional CA, Cyano-Shot™, The Finder™, and LCA) and two dyes (i.e., R6G and Ardrox) were compared [248] – note: given that sebum-rich marks and home-made fuming cabinet without heating and humidity controls were used in this study, the conclusions must be taken with caution and are hence not detailed in this report.

**One-step CA** – The following studies investigated the performance of LCA in different contexts: combined with a portable fuming system [249], compared to the conventional [CA ➭ dye] sequence [250], and integrated into a sequential processing applied to plastic bags [251]. These studies are briefly described here-below, and the most relevant observations/conclusions cited:

- Jones et al. assessed the performance of LCA diffused through a portable fuming system (i.e., LumiFume™) comparatively to a conventional CA cabinet and powder suspensions [249]. In the first step of the study, depletion series of natural fingermarks left on four non-porous substrates (i.e., glass, white tile, black plastic, and laminated wood) and aged up to 28 days were considered. In the second step, a pseudo-operational trial was conducted by collecting three sets of 100 items (i.e., 44 confectionary wrappers, 21 plastic bags, 14 plastic bottles, 14 glass bottles, and 7 cans) from recycling bins. In both steps, the items were processed by one of the three following protocols: [LCA/LumiFume ➭ BY40], [LCA/cabinet ➭ BY40], and powder suspension (BPS or WPS). Overall, the authors concluded that LCA/LumiFume was the most effective protocol (1′469 fingermarks detected during the pseudo-operational trial), followed by LCA/cabinet (1′026) and BPW/WPS (641). The portable fuming system is thought to be used at crime scenes, under a tent, but its higher performance compared to a conventional fuming cabinet is worth being investigated in future studies. The authors also confirmed that the application of BY40 after LCA is recommended on suitable substrates, for it allowed the detection of new fingermarks and improved the quality of already detected ones.
- Farrugia et al. investigated the integration of LCA into a sequential processing applied to plastic bags [251]. Four pseudo-operational trial were conducted in this study. They all relied on the collection and processing of hundreds of plastic carrier bags, from supermarket recycling centres, each one being cut into three each equal parts so that three processes could be compared per trial. The main conclusions were: (1) performing a second LCA fuming cycle, just after the first one, results in more fingermarks being detected; (2) the beneficial impact of BY40 after LCA, in terms of increased fingermark recovery, was especially pronounced in this study, maybe due to a high number of recycled plastic bags; (3) the performance of [CA ➭ BY40] is equivalent to LCA (5%–8%, w/w), but becomes inferior when BY40 is applied after LCA.
- considering various non-porous and semi-porous substrates, Odom et al. compared two sequences: [CA ➭ R6G] and [LCA ➭ R6G] [250]. It should be noted that Kent reacted to this article through a Letter to the Editor, mostly with regards to the use of synthetic pads (i.e., sebaceous oil reference pads) and a methanol-based R6G formulation [252]. Due to the use of unrealistic artificial secretions, the conclusions emitted during this study must be taken with caution and are hence not detailed in this report.

**Sequential process** – The introduction of CA in sequential processes was detailed in other sections of this report: added value of WPS applied in sequence with [CA ➭ BY40] [253] – See Section 4.3.6; determination of the most-suited techniques to be applied on different kinds of adhesives and tapes [254] – See Section 4.4.1; confirmation that the adhesive side of tapes must be protected when processing the non-adhesive side with [CA ➭ R6G] [255] – See Section 4.4.1; determination of the best protocols to detect fingermarks on polymer banknotes [256,257] – See Section 4.4.2; performance of the sequential processing involving LCA and VMD on thermal papers [258] – See Section 4.4.5; impact of CA on the efficiency of protein stains applied subsequently [259] – See Section 4.5.1.

**Use of LWRUV** – Illston-Baggs et al. assessed the performance of LWRUV to observe fingermarks on plastic carrier bags processed with three types of CA (i.e., conventional CA, LCA 10% w/w, and Polycyano) and BY40 [260]. A pseudo-operational trial was conducted by collecting 100 plastic carrier bags from supermarket recycling centres, each one being cut into three each equal parts, one per investigated CA. For all three CA processes, additional fingermarks were observed by using LWRUV, compared to visual examination, but the application of BY40 detected all those marks, in addition to new ones. Overall, the authors concluded that the use of LWRUV is not required if a CA process is followed by the application of a luminescent dye. It should also be noted that [PolyCyano ➭ BY40] led to the highest number of detected fingermarks (217), followed by [CA ➭ BY40] (192) and [LCA ➭ BY40] (147).

**Miscellaneous** – In an extensive study involving natural fingermarks and different storage conditions, CA was recommended over powder and SPR to process fruits and vegetables [261]. Casault et al. compared the performance of four alkyl CA monomers (i.e., methyl, ethyl, *n*-butyl, and 2-octyl), considering three non-porous substrates (i.e., glass, aluminium, and acetate sheets). The authors confirmed that ethyl-2-CA is the most-suited monomer overall [262]. Krelil et al. compared different CA fuming temperatures (i.e., from 50°C to 180°C) to determine the optimal one [263]. A temperature of 120°C provided good results, but the authors observed that the optimal temperature should be 150°C. Further experiments are expected, considering an extended pool of substrates and fingermarks, to confirm this observation.

**Other sections** – The following paper is detailed in another section of this report: use of LCA to detect fingermarks on fabrics [264] – See Section 4.4.3.

**Available review** – Chemically-oriented review about tetrazines and their use in LCA [265].

Acronyms used: **BPS** (black powder suspension), **BY40** (Basic yellow 40), **CA** (cyanoacrylate fuming), **DMAB** (dimethylaminobenzidine), **LCA** (Lumicyano™), **LWRUV** (longwave reflected ultraviolet), **R6G** (rhodamine 6G), **SPR** (small particle reagent), **VMD** (vacuum metal deposition), **WPS** (white powder suspension)

#### 4.3.3 Vacuum metal deposition (VMD)

**Casework** – Lam shared the result of a poll disseminated among Canadian law enforcement agencies and forensic associations to assess the practitioners’ awareness regarding VMD [266]. Overall, most of the participants were aware of the VMD as a fingermark detection technique and agreed that VMD is under-considered in casework. They also reported that the decision to use VMD is driven by the offence committed, rather than by the presence of VMD in a detection sequence. A need for more VMD training was identified, as well as for enhanced communication regarding the availability of VMD in Canada, for several participants were not aware of the status of VMD cabinets currently operational in their country.

**Other sections** – The following papers are detailed in other sections of this report: ability of VMD to detect fingermarks on brass plates and fired cartridge cases [[267], [268], [269], [270], [271]] – See Section 4.4.4; performance of the sequential processing involving VMD and amino acid reagents on thermal papers [272] – See Section 4.4.5; impact of corrosive substances on fingermark recovery [273] – See Section 4.5.3.

#### 4.3.4 Physical developer (PD)

**Formulation** – The replacement of Synperonic N by another non-ionic surfactant has been reported by two groups of researchers [274,275]:

- in the first study, Thomas-Wilson et al. compared different surfactants, from the point of view of their physico-chemical properties and their ability to result in performant PD formulations [275]. At the completion of a multi-experiments study involving several formulations and depletion series of natural fingermarks left on porous substrates, the authors concluded that DGME represents the best candidate to replace Synperonic N. The other surfactants considered in this study were: Brij C10, L23, and S10, Igepal CO-360, polyoxyethylene (10) tridecyl ether, Tergitol 15-S-9, and Tween 20. Tween 20 was discarded due to stability and reliability issues observed under the UK environmental conditions. Despite producing a PD solution comparably performant to Synperonic N, Brij C10 was discarded because its solubilization required heat and the processing time was longer. The other surfactants were quickly discarded for stability and cloudiness issues.
- in the second study, Cartledge et al. conducted a semi-operational study to compare the performance of the newly proposed DGME-based PD formulation with the Synperonic N one [274]. The pseudo-operational trial consisted in processing with PD 662 porous items of different origins (e.g., office papers, envelopes, thermal papers, cardboard – collected from waste bins or provided by people), which were already processed with amino acid reagents three years before. The items were separated in two equivalent batches that were processed with either DGME-based PD or Synperonic N-based one. Overall, the two formulations behaved equivalently: the DGME formulation detected 217 fingermarks (62 previously detected by amino acid reagents + 155 new fingermarks); the Synperonic N formulation detected 200 fingermarks (54 previously detected by amino acid reagents + 146 new fingermarks). With regards to the fingermarks detected by PD only, they represent an increase of 30% with regards to the total number of fingermarks that were previously detected by amino acid reagents. This observation emphasizes the importance of PD in a sequential process. The authors concluded that DGME can be operationally used as an alternative to Synperonic N. The DGME stock detergent has a shelf-life of 12 months, and the PD working solution should be used up to 24 h after its preparation.

**Miscellaneous** – Coulston et al. presented a mechanistic study of the PD process, focusing on the nucleation and growth of silver particles on fingermarks [276]. The authors showed that sub-micron silver particles interact with secretion residue at the early stage of the process, then grow up to 16 μm due to *in situ* silver deposition.

**Other sections** – The following paper is detailed in another section of this report: impact of corrosive substances on fingermark recovery, among which PD [273] – See Section 4.5.3.

Acronyms used: **DGME** (decaethylene glycol monododecyl ether)

#### 4.3.5 Powder dusting

Among the 188 papers associated to powder dusting, 123 papers promoted the dusting of nanoparticles, which represent ca. 65% of the papers dedicated to powder dusting and ca. 30% of all the papers related to fingermark visualisation. In most cases, the fingermark detection capability of the nano-sized powders was a pretext for the synthesis and characterization of optically active nanomaterials. For health and safety reasons, some nanomaterials containing heavy metals such as cadmium or lead, we have decided not to cite any of the 123 papers recommending the powdering of nanoparticles to detect fingermarks.

Most of the papers related to powder dusting were preliminary/pilot studies based on limited sets of sebum-rich fingermarks left on glass or other non-porous substrates. Consequently, an overestimation of the reported performances is expected. Also, for most of these papers, fingermark detection capability is taken as a pretext for the synthesis and characterization of optically active materials. The expressed conclusions are consequently to be taken with extreme caution.

**Preliminary/Pilot studies** – Several compounds were presented as new dusting powders: AIE-active composites or molecules [[277], [278], [279], [280], [281]], algal biomass [282], anti-perovskite phosphors [283], azo-dyes [284], β-cyclodextrin-berberine complexes [285], benzazole derivatives [286], bisamide analogues [287], cadmium-doped silica microspheres [288], chitosan-tripolyphosphate-lysine conjugates [289], coumarin–pyridone conjugates [290], crushed eggshells and clamshells [291], crushed gambir [292], curcumin analogues [293], doped montmorillonite [278,294,295], doped starch microspheres [[296], [297], [298]], europium-dextrose composites [299], iron oxide/zinc sulphide hybrid [300], metal-organic frameworks [301], organic NIR emitters [302,303], perylenetetracarboxylate compounds [304], phenylthiazoles [305], phosphor-cellulose hybrids [306], pyrazoles [307], pyrene-tagged imidazolium salts [308], pyridine and pyrimidine derivatives [309,310], rare-earth-doped phosphors [[311], [312], [313], [314], [315], [316], [317], [318], [319]], reduced graphene oxides [320], resveratrol powder [321], rhodamine B-doped diatomaceous earth [322], Schiff base complexes [[323], [324], [325]], talcum powder [326], triazine-based phosphors [327]. Two papers proposed alternatives to the use of a brush to apply powder: cabinet equipped with fans [328] and low-pressure sandblaster combined with fluorescent starch powder [329].

**Comparative study** – Considering depletion series of natural fingermarks deposited on four non-porous substrates (i.e., glass, aluminium drink cans, PE bags, and ceramic tiles) and aged up to 14 days, Chadwick et al. compared the performance of two NIR powders (i.e., fpNATURAL® 1 and Universal Powder) and two conventional ones (i.e., black and GREENcharge™) [330]. Universal Powder is described as an in-house powder made of Styryl 11, R6G and GREENcharge™ magnetic powder. The main conclusions were: (1) overall, the performances of the two conventional powders and the Universal Powder were similar, (2) NIR imaging did not significantly improve the contrasts, compared to white light or luminescence, even on patterned substrates (i.e., aluminium cans), (3) fpNATURAL® 1 led to the poorest performances on all the substrates, mostly due to adherence issues and better performance obtained with the Universal Powder.

**Composition** – Six commercial black powders (i.e., Lightning black, Supranano black, Lynn Peavey, Evident, Sirchie 101L HiFi Volcano, Arrowhead) were characterized by various analytical techniques (i.e., XPS, SEM, EDS, DLS, ATR-IR, Raman spectroscopy, PXRD, and NMR) [331]. All powders were mostly composed of carbon and oxygen, with traces of other chemicals in varying quantities (e.g., sulphur, silicon, iron, aluminium). Only the Arrowhead powder distinguished itself by a significant amount of manganese (9.1%). In terms of particle size, all the six powders are composed of nano-sized particles, sometimes with average diameters below 50 nm (i.e., Lightning black and Lynn Peavey). Only Evident and Arrowhead powders contained a significant proportion of micron-sized particles. The powders were also ranked by performance, but the proposed ranking must be taken with caution given the use of artificial eccrine and sebum emulsion combined with the absence of clear assessment criteria provided to the assessors – Note: the fact that some commercially-available powders are mostly composed of nano-sized particles, instead of micron-sized particles/flakes historically associated with conventional powders, should raise some concerns from the practitioner community.

**Miscellaneous** – Citing late 19th and early 20th century documents, Claveria proposed to link the discovery of powder dusting to Alphonse Bertillon rather than to Dr. René Forgeot [332]. Black powder was recommended over CA and NIN to detect fingermarks on fruits, vegetables, and snacks [333]. Black magnetic powder was recommended over CA and NIN to detect fingermarks on quail eggshells, although it was determined to be detrimental for subsequent DNA profiling [334]. Nontiapirom et al. showed that genetic material could be present on new fingerprint brushes, and that DNA transfer could occur if the brush is applied on a fresh/wet spot of saliva before dusting fingermarks [335].

**Other sections** – The following papers are detailed in another section of this report: determination of the best protocol to detect fingermarks on polymer banknotes [257] – See Section 4.4.2; use of liquid latex to remove debris from the exterior surfaces of cars before fingermark dusting [336,337] – See Section 4.4.6; use of powders to detect fingermarks on painted surfaces [338] – See Section 4.4.6; processing of non-porous items immersed in water [339] – See Section 4.5.2.

Acronyms used: **AIE** (aggregation-induced emission), **ATR-IR** (attenuated total reflectance infrared spectroscopy), **CA** (cyanoacrylate fuming), **DLS** (dynamic light scattering), **EDS** (energy dispersive X-ray spectroscopy), **NIN** (ninhydrin), **NIR** (near infrared), **NMR** (nuclear magnetic resonance spectroscopy), **PE** (polyethylene), **PXRD** (powder X-ray diffraction), **R6G** (rhodamine 6G), **SEM** (scanning electron microscopy), **XPS** (X-ray photoelectron spectroscopy)

#### 4.3.6 Powder suspensions

**Preliminary/Pilot studies** – *C. Rugosa* lipase [340] and rust fungi combined with zinc carbonate [341] were presented as contrasting agents that could be introduced in powder suspensions. The recording of fingermarks from items processed by powder suspensions could be carried out while maintaining the substrate immersed in clear water [342]. This way of doing was claimed to improve the contrast and the quality of the detected fingermarks, while preserving any sensitive surface from oxidation (e.g., firearm or electronic device).

**Sequential processing** – The added value of a sequential application of WPS, subsequently to CA, has been emphasized by the forensic laboratory of the Provincial Police Headquarters in Szczecin (Poland) [253]. The author reported the results of a study carried out between August 2019 and December 2020, on items related to 288 cases [253]. On foil packaging, plastic items, and substrates for which CA proved to be ineffective, the following sequence has been applied: [CA ➭ BY40 ➭ WPW]. Overall, the total number of *identifiable* fingermarks detected by [CA ➭ BY40] was 375, mostly recovered on glass bottles and drug-related items (e.g., aluminium foil and plastic packaging). WPW applied subsequently detected 255 new fingermarks and enhanced the quality of 65 fingermarks, mostly on items made of plastic and drug-related packaging (e.g., plastic package and stretch foil). This study tends to prove that WPW can be applied in sequence with [CA ➭ BY40], resulting in a substantial number of new fingermarks.

**Other sections** – The following papers are detailed in other sections of this report: new carbon-based BPS to detect fingermarks on the adhesive side of tapes [343] – See Section 4.4.1; determination of the most-suited techniques to be applied on tapes [254] – See Section 4.4.1; confirmation that the adhesive side of tapes must be protected when processing the non-adhesive side with [CA ➭ R6G] [255] – See Section 4.4.1; performance of amino acids reagents to detect fingermarks on different kinds of gloves [240] – See Section 4.4.6; activated-charcoal-based SPR to detect fingermarks on immersed non-porous items [344] – See Section 4.5.2; impact of corrosive substances on fingermark recovery [273] – See Section 4.5.3.

Acronyms used: **BPS** (black powder suspension), **BY40** (Basic yellow 40), **CA** (cyanoacrylate fuming), **R6G** (rhodamine 6G), **SPR** (small particle reagent), **WPS** (white powder suspension), **WPW** (Wet Powder White)

#### 4.3.7 Nanoparticles in solution

**Preliminary/Pilot studies** – Several NPs were proposed to be applied in solution to detect fingermarks on various substrates: BSA-functionalized fluorescent NPs dispersed in poly(vinyl alcohol) [345], carbon nanotubes functionalized with *C. Rugosa* lipase [[346], [347], [348]], carbon QDs-soaked membranes [349], citrate-capped lanthanide-doped upconversion NPs [350], dye-doped PEG-based micelles [351], fluorescent aptamer-functionalized covalent organic framework hydrogels [352], fluorescent carbon NPs [353], lysozyme-specific aptamer-coated gold NPs followed by adhesive transfer and chemical imaging [216], 3-mercaptopropionic-acid-capped cadmium-based NPs and nanorods [354], NIR-emitting core-shell QDs [355], perovskite nanocrystals [356], QD-doped MOF and silver nanocluster nanohybrids [357], R6G-doped gold-palladium core-shell nanorods [358], silica NPs to detect bloody fingermarks [359], silver NPs [360,361], SMD II combined with chemical imaging [220], transfer on polyamide-based nanofibrous membrane combined with cadmium-based QDs [362]. Note: most of these preliminary/pilot studies relying on limited sample sets (e.g., one donor, few depositions, sebum-rich secretions, fresh fingermarks), an overestimation of the reported performances is expected.

**Colloidal gold** – MMD I was recommended over black powder and [CA ➭ black gel lifter] to detect fingermarks on the inside of rubber gloves [363]. The following papers discussed the use of colloidal gold, but are detailed in other sections of this report: MMD I to detect fingermarks on fabrics [364] – See Section 4.4.3; MMD I to process plastic bags made of compostable polymers [365] – See Section 4.4.6; SMD II to detect fingermarks on PE zip-lock bags exposed to stagnant water and outdoor conditions [366] – See Section 4.5.2.

**Silica NPs** – The following papers aimed at optimizing the application of functionalized silica NPs to detect fingermarks: RuBpy-doped carboxyl-functionalized silica NPs [367,368] and upconverter-doped functionalized silica NPs [369,370]. These studies are briefly described here-below, and the most relevant observations/conclusions cited:

- in their first paper, Lee et al. optimized the application protocol of their fluorescent silica NPs and compared their performance to [CA ➭ R6G] [367]. Considering depletion series of natural fingermarks left on six non-porous substrates (i.e., aluminium, PE and PP films, glass, gloss paint, and white ceramic tile) and aged up to six months, the authors made the following observations: (1) overall, the performance of NPs was inferior to conventional methods, (2) silica NPs were especially impacted by the nature of the substrate, but less by the donorship or the secretion quantities. In their second paper, the authors introduced new application protocols (i.e., multiple immersions and spraying) and investigated the possibility to apply silica NPs and [CA ➭ R6G] in sequence [368]. Considering depletion series of natural fingermarks left on three non-porous substrates (i.e., aluminium, PE and PP films) and aged up to 13 months, the authors made the following observations: (1) multiple immersions in silica NPs working solutions could improve the quality of fingermarks without presenting any background staining issue, (2) the possibility to successfully apply silica NPs by spraying has been demonstrated, (3) silica NPs and CA are both detrimental to each other, preventing hence their application in sequence.
- Kanodarwala et al. described the synthesis of carboxyl-capped [370] and phenyl-capped [369] upconverter-doped silica NPs. The first ones led to poor performances when applied on natural fingermarks left on aluminium. The second ones were more promising and were compared to [CA ➭ R6G]. Following a methodology similar to the one described above, the authors observed that the conventional process involving [CA ➭ R6G] was almost systematically superior in performance, regardless of the donors or substrates. However, the authors emphasized the added value brought by upconverters on multicoloured and luminescent substrates, for they can eliminate the background interference. Further experiments are expected on this topic.

**Miscellaneous** – Lanahan and Yoda assessed the ability of carboxyl- and sulfate-terminated polystyrene NPs in an acidic aqueous solution to detect fingermarks on non-porous substrates [371]. Using a so-called “fingerprint press” to decrease the variability of deposition, the authors collected natural and sebum-rich fingermarks on glass, which were then aged up to 435 days. When compared to CA and dry dusting, the polysterene NPs gave promising results. Further studies are hence expected.

**Relevant reviews** – Thorough reviews about the use of NPs to detect fingermarks, including metal and metal oxide NPs, QDs, carbon dots, silica and upconverters NPs [372,373].

Acronyms used: **BSA** (bovine serum albumin), **CA** (cyanoacrylate fuming), **MMD I** (multi-metal deposition I), **MOF** (metal-organic framework), **NIR** (near infrared), **NPs** (nanoparticles), **PE** (polyethylene), **PEG** (polyethylene glycol), **PP** (polypropylene), **QDs** (quantum dots), **R6G** (rhodamine 6G), **RuBpy** (tris-2,20-bipyridyl dichlororuthenium II hexahydrate), **SMD II** (single metal deposition II)

#### 4.3.8 Lipid stains

**Preliminary/Pilot studies** – The following compounds were synthesized and successfully applied to sebum-rich fingermarks: dialkylated NB and NR derivatives [374], purine derivatives [375], and fumed pyrene [376]. The combination of iodine fuming with hexane or chloroform spraying was presented to detect fingermarks on various substrates [377]. Fingermarks left on stainless steel metal plates that were buried in the soil for up to eight weeks were successfully detected using SB [378]. Note: most of these preliminary/pilot studies relying on limited sample sets (e.g., one donor, few depositions, sebum-rich secretions, fresh fingermarks), an overestimation of the reported performances is expected.

**Other sections** - The following paper was detailed in another section of this report: impact of ethanol on the quality and recovery of fingermarks by amino acid reagents and ORO [241] – See Section 4.5.3.

Acronyms used: **NB** (Nile blue), **NR** (Nile red), **ORO** (Oil red O), **SB** (Sudan Black).

#### 4.3.9 Other techniques

Most of the papers cited below were preliminary/pilot studies based on limited sets of sebum-rich fingermarks left non-porous substrates. Consequently, an overestimation of the reported performances is expected. Also, for most of these papers, fingermark detection capability is taken as a pretext for the synthesis and characterization of optically active materials. The expressed conclusions are consequently to be taken with extreme caution.

**Preliminary/Pilot studies** – Several compounds to be applied in solution were proposed to detect fingermarks on various substrates, mostly non-porous: AIE-active molecules or complexes [230,245,[379], [380], [381], [382], [383], [384], [385], [386], [387], [388]], benzothiazole derivatives [389], conjugated cationic polymers in solution [390], di-aqua tris(thenoyltrifluoroacetonate) europium III complex [391], dye-doped amphiphilic conjugated polymer micelles [392], dye-doped octasilsesquioxane hybrids [393,394], phenolic ligands chelated with metal ions [395], sequential processing involving the lifting of fingermarks by a cellulose membrane followed by immersion in water or chemical imaging [218], tetraphene derivatives [396]. Other reagents or mechanisms were also proposed: deposition of metallic films (i.e., copper, chromium, and aluminium) using magnetron sputtering [397], soot from camphor [398], sublimation of 9-fluorenone under vacuum [399].

**Immunodetection** – van Dam et al. recommended a multi-target immunolabeling approach, targeting dermcidin and albumin, to detect fingermarks on non-porous substrates [400]. Targeting keratin led to no added value and was hence not recommended. When combined with conventional fingermark detection techniques (i.e., black magnetic powder and CA), the authors showed that: (1) no or limited detrimental impact was observed on immunolabeling performance if powder or CA were applied first, respectively, and (2) significant detrimental impact to powder and CA was observed if immunolabeling was applied first. In a similar context, a preliminary study presented the use of antibodies combined with a luminol-based chemiluminescence imaging step to detect bloody, natural, and eccrine-rich fingermarks on non-porous substrates [401].

**ESDA** – The possibility to use ESDA to detect fingermarks on porous substrates has been investigated [402]. Considering depletion series of three types of fingermarks (i.e., natural, sebum-rich, and eccrine-rich) left on paper and aged up to 70 days, the authors showed that: (1) sebum-rich marks were quite well detected by ESDA for up to 16 days, (2) eccrine-rich marks were faintly detected overall and presented a reversed contrast, and (3) natural fingermarks of more than one day were poorly detected.

Acronyms used: **AIE** (aggregation-induced emission), **CA** (cyanoacrylate fuming), **ESDA** (electrostatic detection apparatus)

### 4.4 Substrates

#### 4.4.1 Adhesives and tapes

**Comparative studies and sequential process** – Martinez confirmed that the adhesive side of tapes must be protected when processing the non-adhesive side with [CA ➭ R6G], to avoid any detrimental impact on the performance of powder suspension applied subsequently on the adhesive side [255]. A new carbon-based BPS, called “WET UCIO” and containing sodium lauryl sulfate as surfactant, was proposed to detect fingermarks on the adhesive side of rubber-based and acrylic-based tapes [343]. WET UCIO showed equal or higher performance compared to conventional powder suspensions (Wetwop and ASPD), further experiments being expected to confirm these trends. In a tape analysis context, Chadwick et al. investigated the impact of some fingermark detection techniques (i.e., Wet Powder, [CA ➭ R6G] and [CA ➭ Wet Powder]) on the optical and physico-chemical characteristics of pressure-sensitive tapes (e.g., duct, electrical, masking and packaging tapes) [403]. Bradshaw et al. investigated if MALDI-MSI could be applied after conventional techniques (e.g., CA ➭ BY40, BPS, GV, VMD_Au/Zn_) to detect fingermarks on the adhesive-side of tapes [223]. Considering fresh natural fingermarks left on clear and brown tapes, the authors demonstrated that MALDI-MSI could be applied as an end process to different sequences, for example [CA ➭ VMD_Au/Zn_ ➭ MALDI-MSI] and [CA ➭ BY40 ➭ BPS ➭ GV ➭ MALDI-MSI], resulting in well-contrasted images without background contribution.

Garcia and Gokool aimed at determining the most-suited techniques to be applied on duct, electrical (black and blue), packing (brown and clear) and Scotch (opaque) tapes [254]. Using 1-day-old to 6-week-old depletion series of natural fingermarks, the authors compared nine techniques: BPS/Photo-Flo, BPS/Liqui-nox, [CA ➭ BY40], [CA ➭ R6G], GV, Liqui-Drox, SSP, TapeGlo, and Wetwop. The most suitable techniques associated to each tape were the following: electrical tapes = Wetwop, BPS/Liqui-nox, or Liqui-Drox; brown packing tape = Wetwop; clear packing tape = SSP; duct tape and Scotch = Wetwop. [CA ➭ BY40] and [CA ➭ R6G] were also considered as second-most suited techniques for black electrical tape (BY40), clear packing tape (BY40), duct tape (R6G), and Scotch (R6G). The authors emphasized the fact that these results are limited to the tapes and substrates considered in this study. They also recommend testing evidentiary items for background staining before applying any technique.

**Adhesive removal** – A 30% HFE-72DE:HFE-7200 (v/v) solution is proposed to remove duct tapes from porous substrates, with minimal impact on fingermarks and higher performance compared to Un-Du [404].

Acronyms used: **ASPD** (adhesive-side powder dark), **BPS** (black powder suspension), **BY40** (Basic Yellow 40), **CA** (cyanoacrylate fuming), **GV** (gentian violet = basic violet 3), **MALDI-MSI** (matrix-assisted laser desorption ionization mass spectrometry imaging), **R6G** (rhodamine 6G), **SSP** (sticky-side powder), **VMD**_**Au/Zn**_ (gold/zinc-based vacuum metal deposition)

#### 4.4.2 Banknotes

Three studies aimed at proposing the best protocol to detect fingermarks on banknotes: NIS 100 and NIS 200 [256], £5 and £10 Clydesdale Bank and Royal Bank of Scotland [257], and RMB [224]. The most relevant observations/conclusions of these studies were the following:

- on NIS 100 and NIS 200 polymer banknotes, [CA ➭ RUVIS@254 nm] was determined to be the most-suited detection sequence [256]. The other detection techniques that were considered in this study were VMD_Au/Zn_ and black magnetic powder. Further experiments are expected, to assess the added value of extending this sequence with additional fingermark detection techniques.
- on £5 and £10 Clydesdale Bank and Royal Bank of Scotland polymer banknotes, [PolyCyano UV ➭ black magnetic powder] combined with IR observation and recording (730–800 nm) was determined to be the most-suited detection sequence [257]. The other detection techniques that were considered in this study were fpNatural 2 and BPS. Further experiments are expected, to assess the performance of this sequence on other polymer banknotes as well as on circulated banknotes.
- the proof-of-concept of imaging fingermarks on worn RMB cotton-based banknotes using ToF-SIMS has been presented [224]. Due to the limited size of the scanning area, banknotes had to be folded.

Acronyms used: **BPS** (black powder suspension), **CA** (cyanoacrylate fuming), **IR** (infrared), **NIS** (new Israeli shekel, IL), **RMB** (renminbi, CN), **RUVIS** (reflected UV imaging system), **ToF-SIMS** (time of flight secondary ion mass spectroscopy), **UV** (ultraviolet), **VMD**_**Au/Zn**_ (gold/zinc-based vacuum metal deposition)

#### 4.4.3 Fabrics

Four papers investigated the performance of different techniques to detect fingermarks on fabrics: LCA [264], IND/Zn [239], MMD I [364], and flash overexposure [205]. These studies are briefly described here-below, and the most relevant observations/conclusions cited:

- LCA with 9% (w/w) dye showed good performance to detect fingermarks on different types of fabrics (i.e., polyester, silk, cotton, satin, nylon) [264]. The subsequent staining with a luminescent dye (i.e., BY40) was not recommended. The other detection techniques that were considered in this study were conventional CA and BY40 staining. Further experiments are expected, including more fabrics, fingermark donors and aging times.
- Galinsky et al. assessed the possibility to spray IND/Zn to locate grabbing areas on fabrics [239]. Considering four types of fabrics (i.e., cotton denim, cotton-polyester denim, cotton, and polyester) and fingermarks aged up to seven days, the authors showed that IND/Zn successfully detected grab areas on 86% of the samples. The best results were obtained with cotton-polyester denim, followed by cotton denim, polyester, and cotton. After spraying IND/Zn, the fabrics could either be placed in a humidity chamber (i.e., 70°C, 65% RH) for 20 min, or left to develop under room conditions for 2 h. Note: ridge details have not been discussed by the authors, but it seems that only grabbing area presenting no ridge details were observed.
- the ability of MMD I to detect fingermarks on different types of light-coloured fabrics (i.e., nylon, polyester, satin, pongee, cotton, and oxford) was investigated [364]. MMD I showed good performance on light-coloured, smooth and tightly weaved textiles of artificial origin (as opposed to oxford and cotton). The impact of the aging times (up to 28-day-old fingermarks) and of the immersion of the items prior to fingermark detection (up to 84 h in water) was also investigated. The other detection techniques that were considered in this study were MMD II and hybrid MMD formulations.
- Rimmasch proposed to intentionally overexpose dark-coloured fabrics up to +3 EV to improve the visualisation of wet and blood marks [205]. The performance of this approach is not comparable to IR imaging but may constitute a useful alternative for laboratories not equipped with IR imaging capabilities.

Acronyms used: **BY40** (Basic Yellow 40), **CA** (cyanoacrylate fuming), **EV** (exposure value), **IND/Zn** (1,2-indanedione combined with zinc chloride), **IR** (infrared), **LCA** (Lumicyano™), **MMD I/II** (multi-metal deposition I/II), **RH** (relative humidity)

#### 4.4.4 Metal and cartridge cases

**Preliminary/Pilot studies** – Redox approaches were proposed to detect fingermarks on cartridge cases or other metallic surfaces: aryldiazonium tetrachloroaurate as redox agent to detect fingermarks on copper [405] or nickel coins [406], deposition of cuprous sulphide on unfired brass cartridges [407]. The electrodeposition of polymers was also proposed to detect fingermarks on various metal surfaces, sebum-rich secretions acting as mask preventing the polymerization process: polycaprolactone nanofibers on aluminium, stainless steel, and cartridges [408], poly(3,4-ethylenedioxythiophene) on unfired brass cartridges [409], polyluminol on indium tin oxide coated glasses [410], poly(neutral red) on platinum and brass [411,412], polypyrrole combined with poly(2,2′:5′,2″-terthiophene) on stainless steel [413], and polypyrrole derivatives on stainless steel [414]. Other electrodeposition processes were proposed: cobalt oxide films on stainless steel [415], copper on aluminium [416], nickel/phosphor composites on copper [417]. In a chemical imaging context, Lee et al. presented a rotation device used in combination with ToF-SIMS to flatten the fingermarks left on cartridge cases [221]. Note: these preliminary/pilot studies relying on limited sample sets (e.g., one donor, few depositions, sebum-rich secretions, fresh fingermarks), an overestimation of the reported performances is expected.

**Disulfur dinitride (S**_**2**_**N**_**2**_**) –** S_2_N_2_ is a recent reagent aiming at detecting fingermarks on metal through exposure to fumes in a sealed cabinet. Only one commercial device currently allows the use of this technique: RECOVER® (Foster + Freeman). Three studies aimed at assessing the performance of S_2_N_2_ to detect fingermarks on metal plates [418] and fired brass cartridge casings [419,420]. These studies are briefly described here-below, and the most relevant observations/conclusions cited:

- Bleay et al. conducted a series of experiments involving the detection of fingermarks on different metal plates (i.e., brass, bronze, copper, and stainless steel), using five detection techniques (i.e., S_2_N_2_, [CA ➭ BY40], [VMD_Au/Zn_ ➭ VMD_Ag_], GB and BPS) and depletion series of natural fingermarks [418]. Adverse conditions (i.e., water/detergent washing and scrubbing, acetone washing, and exposure to 600°C for varying times) and different ageing times (i.e., from 1-day-old to 3-month-old fingermarks) were also considered. This study was conducted before the RECOVER® device (Foster + Freeman) was commercially available, using hence an in-house device. Numerous observations could be found in the paper, the most relevant conclusions being: (1) S_2_N_2_ consistently allowed the visualisation of good quality marks over the different experiments; (2) in non-detrimental conditions, [CA ➭ BY40] and S_2_N_2_ proved to be highly sensitive on brass and developed the highest number of high-quality marks; (3) stainless steel proved to be the most challenging substrate, especially for GB which could not detect any mark on it; (4) VMD and S_2_N_2_ were the least impacted by the age of the fingermarks, as opposed to GB and CA, whose performances decreased with the fingermark age; (5) several fingermarks processed by [CA ➭ BY40] were not visible by eye. Surprisingly, fingermarks showed a non-negligible level of resilience against the water/detergent and acetone washing steps, for some of them could still be developed by the different detection techniques (with varying success rates). In that context, the older the marks, the higher the recovery rate post-washing. It is hypothesized by the authors that the corrosion initiated by some of the fingermark components resulted in local surface modifications which survive the washing event and induce a difference of reaction between the fingermark location and the surrounding substrate.
- Wilkinson et al. investigated the impact of different parameters, among which the item storage and the cleaning protocol, on the performance of S_2_N_2_ to detect fingermarks on fired cartridges or exploded devices [419]. The authors considered depletion series of natural fingermarks left on brass cartridges that were fired afterwards, as well as sebum-rich fingermarks left on brass plates that were subsequently used in an IED. The authors emphasized the importance of avoiding storage in a hot and humid environment. They also emphasized the importance of the cleaning protocol, to remove any residue which may initiate the unwanted polymerization of S_2_N_2_. Scrubbing the item bearing fingermarks with warm soapy water followed by acetone rinsing is hence mandatory for this detection technique, even if it appears counter-intuitive with regards to fingermark preservation and conventional recovery processes. When considering ambient temperature storage and the recommended cleaning protocol, the results were the following: of the 153 fingermarks that were left on brass cartridges that were subsequently fired, 34 (22%) were of sufficient quality for comparison, among which 16 (10%) were *identifiable.* A significant impact of the age of the fingermarks upon firing has been emphasized, a drop in the success rates being observed for one-week-old fingermarks compared to one-day-old ones. Further experiments are expected, to further explain the underlying mechanisms and investigate the introduction of the technique in sequence with conventional ones.
- Pontone et al. assessed the performance of the RECOVER® device on spent brass cartridge cases [420]. The study focused on three experiments: (E1) detection of natural and sebum-rich fingermarks using RECOVER® only, (E2) detection of sebum-rich fingermarks via [CA ➭ R6G ➭ RECOVER®], and (E3) impact of DNA swabbing prior to RECOVER®. All fingermarks were 3-day-old when the cartridges were fired. Overall, 40% of the deposited fingermarks (i.e., 157 of 390) were observed after the RECOVER®, but only 1.5% (i.e., 6) were suitable for comparison. The conclusions of the different experiments were the following: (E1) 23% of the fingermarks were detected, but only 2% (two sebum-rich fingermarks) were suitable for comparison, (E2) 23% of the fingermarks were observed after [CA ➭ R6G], but none were suitable for comparison; for RECOVER®, the recovery and comparison rates were of 34% and 1%, respectively; (3) dry or wet DNA swabbing had no detrimental impact on the subsequent fingermark detection by RECOVER®.

**Gun Blue –** One paper investigated the ability for GB (50% v/v) to detect fingermarks on brass plates and cartridges [421], and a two-part study further investigated the eGB process [422,423]. These studies are briefly described here-below, and the most relevant observations/conclusions cited:

- Christofidis et al. investigated the ability of GB (50% v/v) to detect sebum-rich and natural fingermarks on brass plates and on fired brass cartridges, considering different aging times (up to 30 days) and various storage conditions (i.e., dark, indoor, outdoor) [421]. No optimal processing time could be recommended, for it ranged from 10 to 30 s for marks left indoor to 100–150 s for marks left outdoor for one month.
- the eGB process consists in applying an electric charge to the metal during the exposure to GB, as opposed to a passive GB reaction. Dove conducted a study assessing the influence of the fingermarks (i.e., composition, donors, age, depletion series – considering brass cartridges and plates) and of the substrates (i.e., nickel-plated and steel cartridge cases – fingermarks being placed after the cartridges were fired) [422]. eGB performed quite well with regards to fingermark composition (especially sebum-rich and natural marks), depletion series, and aging times up to 10 weeks. For nickel-plated cartridges, eGB showed promising results compared to passive GB and the author recommended adapting the experimental setup for this kind of substrate (i.e., 15% v/v solution, 4.5 V). For brass, the author recommended a 15% (v/v) GB solution and a 1.5V potential. On steel cartridges, neither eGB nor passive GB were recommended, mostly due to contrast and reactivity issues. - in a follow-up study, Dove et al. investigated the impact of eGB on the toolmarks, considering brass cartridges, as well as the impact of eGB, passive GB, and palladium deposition on the genetic material (DNA), considering diluted blood left on brass plates [423]. The results showed that eGB has a noticeable negative impact on faint toolmarks. The authors hence recommended conducting toolmark examination prior to fingermark detection using eGB. About the genetic material, all the techniques had a negative impact on the DNA analysis when using the Promega DNA IQ system. If some profiles could be obtained with passive GB and palladium deposition, no profile could be obtained from the samples processed with eGB. One explanation may be that this technique had a major negative impact on the DNA extraction step. The authors consequently did not recommend to currently apply eGB in combination with toolmark examination and DNA analysis, if eGB is to be applied first.

**VMD** – A series of preliminary studies (few donors and/or sebum-rich fingermarks) investigated the ability of VMD to be applied on brass plates and fired cartridge cases [[267], [268], [269], [270], [271]]. Given their preliminary nature, all these studies require further experiments to strengthen their conclusions, which were the following:

- positive results were obtained with VMD_Au/Zn_ and VMD_Ag/Zn_ [268,270], both studies emphasizing the influence of the donor and the detrimental impact of the firing event.
- In her comparative studies, Brewer concluded that VMD_Al_ suited best brass rifle cartridge casings [267], VMD_Au/Zn_ – wad shotgun cartridges [267], and VMD_Ag_ – brass handgun casings [269]. The other VMD processes considered in Brewer's studies were: [silver ➭ zinc], sterling silver and [copper ➭ zinc].
- Shipman compared the performance of VMD_Au/Zn_ and VMD_Ag/Zn_ with [CA ➭ BY40 or R6G] on different items, including handguns (i.e., Glock 19 and Beretta) and unfired brass ammunitions [271]. On the handgun, VMD_Ag_ provided the best results, followed by [CA ➭ R6G]. On brass cartridges, both VMD processes overperformed [CA ➭ BY40]. The author emphasized the difficulty to process a multi-surface item, such as a handgun, with VMD_Au/Zn_ as it resulted in heterogeneous metal deposition.

**Case reports** – Two papers emphasized the importance of preliminary visual examination of firearm-related items for any visible friction ridge details [424,425]. Two other papers reported fingermark recovery rates on firearm-related items [426,427]. These studies are briefly described here-below, and the most relevant observations/conclusions cited:

- Dove described a 2016 RCMP case during which 25 handguns, 25 magazines and 98 loaded cartridges were seized from an individual [424]. Upon visual examination, friction ridge details were observed on three separate cartridges. After processing the items with [CA ➭ R6G], an unwanted polymerization over the surfaces prevented any further observation of friction ridge details.
- at a homicide scene in Kansas City, four cartridges were collected and swabbed for DNA [425]. When reviewing the pictures taken at the crime scene, it was noticed that one of the cartridges presented friction ridge details. Upon verification of the cartridge, it appeared that the fingermark survived the swabbing process and was eventually associated to the suspect.
- Koning reported the recovery rates associated to 2′000 cases processed in 2017 and 2018 by the Colorado Bureau of Investigation – Forensic Services laboratories (i.e., 500 cases per laboratory) [426]. The sequence associated to non-porous items was the following: [visual examination ➭ CA ➭ dye]. The recovery rates associated to firearm-related items were the following: handguns (4.34%), long guns (24.07%), magazines (9.45%), fired cartridge cases (0.38%), and live cartridge cases (0.37%). Some other recovery rates, for items not related to firearms, are reported in Section 4.3.6.
- Swank and Davis reported the fingermark recovery rates associated to the 2017 Las Vegas shooting case [427]. In that case, 6′909 live cartridges, 186 magazines, 6 shotguns, 1 revolver, 5 pistols, and 30 rifles were collected and processed for fingermarks using the following sequence: [visual examination (different wavelengths) ➭ CA ➭ RUVIS ➭ RAM]. It should be noted that the loaded magazines were processed with CA prior to cartridge removal. After their removal, the cartridges were processed through the full sequence (including CA). The recovery rates were the following: firearms (12%), magazines (9.7%), live cartridges (0.38%). It should also be noted that most fingermarks were observed by the application of [CA ➭ RAM]. None of the fingermarks were identified to the gunman and two fingermarks were associated to an individual working at the factory manufacturing the magazines.

Note: in these last two papers, the recovery rates were determined by dividing the number of items bearing at least one fingermark of value [426] or suitable for comparison [427] by the total number of processed items. The reported figures were comparable to the recovery rates that can be found in the literature.

**Miscellaneous** – King and Davis proposed to use of a transparent low-tack adhesive polyester film to protect the headstamp of a cartridge case when processing it for fingermarks [428]. The proof-of-concept was demonstrated using S_2_N_2_ as fingermark detection technique, the protected area being not modified by the reagent.

Acronyms used: **BPS** (black powder suspension), **BY40** (Basic Yellow 40), **CA** (cyanoacrylate fuming), **eGB** (electrodeposited gun blue), **GB** (gun blue), **IED** (improvised explosive device), **R6G** (Rhodamine 6G), **RAM** (R6G, Ardrox, and 7-[pmethoxybenzylamino]-4-nitrobenz-2-oxa-1,3-diazole), **RCMP** (Royal Canadian mounted police), **RUVIS** (reflected UV imaging system), **ToF-SIMS** (time of flight secondary ion mass spectroscopy), **UV** (ultraviolet), **VMD**_**Ag**_ (silver-based vacuum metal deposition), **VMD**_**Al**_ (aluminium-based vacuum metal deposition), **VMD**_**Ag/Zn**_ (silver/zinc-based vacuum metal deposition), **VMD**_**Au/Zn**_ (gold/zinc-based vacuum metal deposition)

#### 4.4.5 Thermal papers

**Preliminary/Pilot studies** – The addition of liquid glue to a NIN solution (0.02–0.05 g/ml) was claimed to prevent the darkening of thermal papers while allowing NIN to react with fingermarks [231]. Exposure to nitrogen dioxide fumes, generated *in situ* by the reaction of nitric acid and zinc, is presented as a solvent-free method to detect fingermarks on thermal papers [429]. Note: these preliminary/pilot studies relying on limited sample sets (e.g., one donor, few depositions, sebum-rich secretions, fresh fingermarks), an overestimation of the reported performances is expected.

**Collaborative exercise** – Organized in the frame of the 2018 EFP-WG activities, 47 laboratories conducted a CE during which they were asked to process a toll ticket for fingermarks, using the procedure they usually apply to thermal papers [430]. The authors detailed the way the CE was created, how the results were assessed, as well as a detailed analysis of the gathered results. The main conclusions were: (1) preliminary observation using a forensic light source and filters is highly recommended as it allows observing latent fingermarks prior any chemical treatment, (2) a sequential processing combining different detection techniques is highly recommended as it allows improving the visibility of some fingermarks, and (3) the gathered data did not support the processing of thermal papers by CA, powders, or iodine. It should be noted that eccrine fingermarks were used in this CE, in addition to uncontrolled fingermarks which may have naturally been on the toll tickets. The behaviour of eccrine secretions may differ significantly from natural fingermarks.

**Visual examination** – Cappiello et al. assessed the interest for NIR luminescence when observing latent fingermarks on the thermal side of thermal papers, prior any chemical treatment [431]. In a pseudo-operational trial, 357 toll tickets, collected three years before, were observed using UV illumination (365 nm) combined with a long-pass observation filter (693 nm). Fingermarks of good quality were observed on 53 tickets (15%). Under this optical configuration, the fingermarks appeared as dark ridges on a luminescent background.

**Sequential process –** Three papers assessed the performance of sequential processes involving: LCA and VMD [258], VMD and amino acid reagents [272], HPS, NIN and ThermaNIN [432]. These studies are briefly described here-below, and the most relevant observations/conclusions cited:

- using depletion series of one-week-old natural fingermarks, Sherriffs et al. first reached the conclusion that [LCA 8% (w/w) ➭ VMD_Au/Zn_ ➭ VMD_Ag/Zn_] was the optimum sequence to process thermal papers [258]. However, they reported that LCA had a detrimental impact on the subsequent VMD_Au/Zn_ processing, and that most of the sequence performance came from VMD_Ag/Zn_. The authors then showed that VMD should be used as a stand-alone technique, rather than in a sequential process involving LCA. In this context, the authors emphasized the influence of the thermal paper brand and of the quantity of metal on the performances of VMD_Au/Zn_ relative to VMD_Ag/Zn_. Further studies are awaited.
- in a pseudo-operational study involving 150 receipts that were cut in half, Illston-Baggs et al. compared two sequences: Seq1/[R–UV ➭ VMD_Au/Zn_ ➭ VMD_Ag_ ➭ acetone bath ➭ IND/Zn ➭ NIN] and Seq2/[R–UV ➭ acetone bath ➭ IND/Zn ➭ NIN] [272]. The acetone bath removes the thermal layer and prevents any darkening caused by amino acid reagents. Overall, the authors recommended to combine VMD with amino acid reagents, for Seq1 led to the detection of +5% (thermal side) and +26% (back side) fingermarks compared to Seq2. Additional fingermarks were obtained at each step of Seq1; in details: +120% (thermal) and +108% (back) fingermarks detected by IND/Zn, compared to VMD; +21% (thermal) and +12% (back) fingermarks detected by NIN, compared to IND/Zn. Other relevant observations were made: (1) R–UV is particularly adapted to the visualisation of latent fingermarks on the thermal side prior any chemical treatment; (2) any printed text should be recorded before processing the thermal papers, for the acetone bath removes it. Further experiments are expected, considering one VMD process instead of two.
- in a pseudo-operational study involving 1′000 receipts that were cut in three parts, Robb et al. assessed the performance of HPS, [acetone bath ➭ NIN], and ThermaNIN, applied alone or in sequence [432]. Overall, the most efficient process was [acetone bath ➭ NIN]. As for the previous study, the acetone bath aimed at removing the thermal layer and preventing any darkening of the item upon application of NIN. Other relevant observations were made: (1) R–UV is particularly adapted to the visualisation of latent marks on the thermal side prior any chemical treatment, for 10% of the fingermarks were solely detected by R–UV; (2) the use of HPS is not recommended due to poor performances compared to the other techniques, several troubleshooting events preventing the device to work properly, and a process limited to only one side of the thermal papers. Further experiments are expected, including the use of IND/Zn and PD.

Acronyms used: **CA** (cyanoacrylate fuming), **CE** (collaborative exercise), **EFP-WG** (ENFSI Fingerprint working group), **HPS** (hot print system), **IND/Zn** (1,2-indanedione combined with zinc chloride), **LCA** (Lumicyano™), **NIN** (ninhydrin), **NIR** (near infrared), **PD** (physical developer), **R–UV** (reflected ultraviolet), **VMD**_**Ag**_ (silver-based vacuum metal deposition), **VMD**_**Ag/Zn**_ (silver/zinc-based vacuum metal deposition), **VMD**_**Au/Zn**_ (gold/zinc-based vacuum metal deposition)

#### 4.4.6 Other substrates

Various studies investigated the detection of fingermarks on (un)common substrates. Most of these preliminary/pilot studies relying on limited sample sets (e.g., one donor, few depositions, sebum-rich secretions, fresh fingermarks), an overestimation of the reported performances is expected.

**Animals** – Black magnetic powder was recommended over CA and NIN to detect fingermarks on quail eggshells, despite being detrimental to DNA profiling [334]. Black gel lifters showed promising results in lifting latent (natural) fingermarks on pangolin scales [433].

**Cars** – The use of liquid latex as a pre-treatment to remove debris from the exterior surfaces of cars prior fingermark detection was investigated [336,337]. The procedure consisted in (1) applying three layers of liquid latex on the area of interest, (2) waiting for the latex to dry before removing it, (3) for painted surface only, spraying a gentle mist of water on the area and letting it dry, and (4) dusting fingermarks using black or white powder. The preliminary study focused on the painted surfaces [336]. The authors deposited sebum-rich fingermarks at the bottom of the vehicles to promote the deposition of debris, while recognizing that fingermarks are normally not recovered from this area. Keeping in mind that the experimental setup may differ from casework conditions, promising results were obtained, with 65.5% of fingermarks recovered when debris were removed by latex, compared to 13.3% for surfaces readily dusted for fingermarks. The second experiment focused on the exterior glass surfaces and still considered sebum-rich fingermarks [337]. The authors showed that better results were obtained when these surfaces were readily dusted (68% of fingermarks recovered), compared to the protocol involving the latex pre-treatment (32%). Latex pre-treatment is hence not recommended for glass surfaces. Further experiments are expected.

**Cell phones** – To assess the impact of fingermark detection techniques on the integrity of cell phones (e.g., access to their content), Papamitrou conducted a study using mostly iPhone devices [434]. The author concluded that the following processes had no detrimental impact on the phones: [CA ➭ Ardrox/spray or brush], [CA ➭ MBD/spray or brush], AB, phloxine B, SB, and [VMD_Au/Zn_ ➭ VMD_Ag_]. After the processing, the phones were powered on again and their content checked (e.g., pictures, applications, access to Internet, calls and messages history). Despite some leak observed under the screen with MBD and SB, all the devices were fully operational. It should be noted that the author did not describe if precautions were taken before the devices were powered up (e.g., drying time). Such information would have been relevant for the phones processed with techniques involving immersion steps (e.g., AB, SB).

**Coated printing paper** – Fingermarks on copperplate paper were successfully detected through electrochromism of 1,1′-bis(3-sulfonatopropyl) viologen, absorbed in the paper [435].

**Compostable polymers** – Organized in the frame of the 2020 EFP-WG activities, 40 laboratories conducted a CE during which they were asked to process a plastic bag made of compostable polymers for fingermarks, using the procedure they usually apply to such items [365]. The authors detailed the way the CE was created, how the results were assessed, as well as a detailed analysis of the gathered results. The main conclusions were: (1) preliminary observation using a forensic light source and filters is highly recommended as it allows observing latent fingermarks prior any chemical treatment, (2) the authors of the CE considered MMD I as the reference technique for this substrate, (3) 80% of the participants applied CA first, followed by powdering, dye staining, VMD, or a combination of those, with varying degrees of success, and (4) the gathered data did not support the processing of compostable polymers by amino acid reagents. It should be noted that eccrine, sebum-rich, and hand-cream-enriched fingermarks were used in the CE.

**Computer devices** – Fulea-Magarit considered the processing of keyboard and mouse for fingermark detection, using CA and powder dusting [436]. Their methodology involved a controlled deposition of fingermarks on clean devices, as well as the processing of naturally used devices. The authors underlined: (1) the detrimental impact of the substrate roughness on the quality of fingermarks, (2) the background staining caused by accumulated material on the devices that were naturally used for months, (3) the distortion that may be observed on curved surfaces, and (4) the possibility to detect fingermarks on the underside of keyboards.

**Food** – Black powder was recommended over CA and NIN to detect fingermarks on daily food (e.g., fruits, vegetables, snacks) [333]. In an extensive study involving natural fingermarks and different storage conditions, CA was recommended over powder and SPR to process fruits and vegetables [261]. A sequential process involving the lifting of fingermarks by a cellulose membrane, followed by application of fruit juice to create a recordable contrast was presented to detect fingermarks from fruits and vegetables [219].

**Painted walls** – Black magnetic granular powder was recommended over magneta flake and NIN to detect fingermarks on painted surfaces, considering four different types of paints (i.e., matt, silk, bathroom and eggshell) [338]. The authors also conducted SEM observations to characterize and distinguish matt and non-matt paints.

**Rubber gloves –** In a study based on eccrine fingermarks aged up to 6 weeks, MMD I was recommended over black powder and [CA ➭ black gel lifter] to process the inside of rubber gloves [363]. In a study based on natural fingermarks left inside different type of gloves and aged up to one month, Rousseau et al. recommend the use of (1) BPS and WPS on nitrile and latex gloves, and (2) HFE-700-based IND/Zn on vinyl gloves [240].

**Self-adhesive stamps** – Ruprecht et al. proposed to use the position of the fingermarks transferred from a stamp to the underlying envelope to locate the contact area and locally process the stamp for DNA [437]. In their study, the authors showed that: (1) stamps can be safely removed by using a cyclohexane:propanol (1:1 v/v) solution, (2) more ridge details were observed on the fingermarks transferred to the envelopes (processed with IND/Zn), compared to the fingermarks detected on the stamps (processed with BPS), (3) contact areas with a fingertip were emphasized for 88% of the stamps, at the edges (73%) or elsewhere over the stamps (15%), (4) the method of choice for DNA profiling was the directed fragment approach, which consists in cutting the stamps by following the contact area.

Acronyms used: **AB** (amido black), **BPS** (black powder suspension), **CA** (cyanoacrylate fuming), **CE** (collaborative exercise), **EFP-WG** (ENFSI Fingerprint working group), **IND/Zn** (1,2-indanedione combined with zinc chloride), **MBD** (7-[pmethoxybenzylamino]-4-nitrobenz-2-oxa-1,3-diazole), **MMD I** (multi-metal deposition I), **NIN** (ninhydrin), **SB** (Sudan black), **SEM** (scanning electron microscopy), **SPR** (small particle reagent), **VMD**_**Ag**_ (silver-based vacuum metal deposition), **VMD**_**Au/Zn**_ (gold/zinc-based vacuum metal deposition), **WPS** (white powder suspension)

### 4.5 Contextual situations

#### 4.5.1 Blood-containing fingermarks

**Preliminary/Pilot studies** – Various new reagents or approaches were proposed to detect blood marks on non-porous substrates: conjugated cationic polymers in solution [390], cotton pads soaked with blood reagents [438] or with a fluorescent amino-functionalized conjugated polymer [439], *Laccifer lacca* in solution [440], phenolic ligands chelated with metal ions [395], and silica nanoparticles suspended in ethanol [359]. Acidified hydrogen peroxide combined with 5-sulfosalicylic acid was shown to induce photoluminescence of blood on various porous and non-porous substrates [441]. The local application of diluted EDTA on sebum-rich fingermarks covered with blood was proposed to remove the blood layer and expose the underlying fingermark, which can then be detected using conventional powders or chemical imaging [215]. Exposing blood fingermarks to heat (e.g., 140°C or 225°C from 15 min to an hour) was proposed as an alternative to conventional fixative solutions (e.g., 5-sulfosalicylic acid) before the application of blood reagents [442] – note: the results are highly substrate-dependant, and the authors did not investigate the impact of this protocol on DNA.

**Sequential process** – Bouwmeester et al. investigated the impact of CA on the efficiency of protein stains applied subsequently, more specifically AB (methanol formulation) [259]. The study focused on depletion series of blood fingermarks left on stainless steel plates, aged up to six months, and processed with either [CA ➭ BY40 ➭ AB], [CA ➭ AB] or AB only. The main conclusions were: (1) the application of CA led to an unwanted background staining of AB, (2) the use of BY40 had no detrimental impact on the quality of blood fingermarks, (3) CA improved the ability to retrieve DNA from older blood marks. Further experiments are expected, to confirm these trends.

**Impact on DNA** – Harush-Brosh et al. emphasized a peculiar phenomenon linked to the application of AB (WEAA formulation) on blood fingermarks left on painted plaster wall surfaces [443]. In their experiments, the authors showed that AB partially inhibited the recovery of DNA from blood marks, whereas no such inhibiting effect was observed on non-bloody fingermarks. The same experiment was conducted with NIN, but no such trend was observed. The authors indicated that the behaviour of AB on blood marks may be of use in case of major-to-minor DNA profiles recovered from blood-contaminated fingermarks.

**Blood profiling** – Two studies described the use MALDI-MS on blood marks, to provide intelligence about their human nature [225] and the haemoglobin variants in presence [175]. These studies are briefly described here-below, and the most relevant observations/conclusions cited:

- in the first study, MALDI-MS was applied to blood marks left on a painted surface to (1) provide information about the human nature of the blood and (2) image the distribution of haemoglobin along the ridge pattern [225]. Painted aluminium sheets were used as simulated painted walls, and blood marks were enhanced with NIN or AB (WEAA formulation) before being analysed with MALDI-MS. The study emphasized (1) the influence of paint constituents on the MS analysis, (2) the importance of a homogeneous matrix deposition to obtain ridge details, (3) the possibility to detect haemoglobin from latent blood marks covered with paint, and (4) the possibility to conduct DNA profiling on MALDI-MS-processed marks. The authors proposed an updated workflow for the processing of blood marks retrieved on painted surfaces, involving the use of MALDI-MS instead of presumptive tests (e.g., Kastle-Meyer or Hexagon kit).
- in the second study, blood marks bearing haemoglobin variants were deposited on glass slides and further processed with AB (methanol formulation) before being analysed with MALDI-MS [175]. The authors successfully identified and mapped the haemoglobin variants, while emphasizing that high-end equipment was required to distinguish such variants. Further experiments are expected.

**Case report** – Lin et al. described a case in which a non-bloody fingermark on a firearm got enhanced by the blood in which the pistol was lying [444]. The authors conducted an experiment aiming at reproducing the phenomenon, with success.

**Miscellaneous** – Zar-Pro fluorescent blood lifters were successfully applied to blood fingermarks, semen and saliva stains left on various non-porous, semi-porous and porous substrates [445], but failed in lifting sebum-rich fingermarks. The impact of dry-cleaning and laundering on the visualisation and enhancement of (one-week-old) blood stains was investigated by Tanner et al. [446]. The study included the use of light- and dark-coloured fabrics (i.e., 100% cotton, 100% merino wool, and 60-40% cotton-polyester), Polilight, visible and IR photography, and two blood reagents (i.e., AB and HR, applied on dark- and light-coloured items, respectively). The main conclusions were: (1) the recovery of blood stains was higher with dry-cleaning compared to laundering, with 75% and 33% of the fingermarks still visible afterwards, respectively; (2) cotton and cotton-polyester retained blood better than wool, and (3) blood reagents outperformed visual observation of blood stains, especially AB which detected 100% of the marks on the dry-cleaned cotton and cotton-polyester items.

Acronyms used: **AB** (amido black), **BY40** (Basic yellow 40), **CA** (cyanoacrylate fuming), **EDTA** (ethylenediaminetetraacetic acid), **HR** (Hungarian red), **MALDI-MS** (matrix-assisted laser desorption ionization mass spectrometry), **NIN** (ninhydrin), **WEAA** (water – ethanol – acetic acid)

#### 4.5.2 Immersed items

**Preliminary/Pilot studies** – The following reagents were proposed to detect fingermarks on immersed items (various water types): suspension of *C. Rugosa* lipase [340,[346], [347], [348]], activated-charcoal-based SPR [344]. These preliminary/pilot studies relying on limited sample sets (e.g., one donor, few depositions, sebum-rich secretions, fresh fingermarks), an overestimation of the reported performances is expected.

**PE zip-lock bags** – D'Uva et al. investigated the performance of SMD II and black powder dusting on PE zip-lock bags exposed to stagnant water and to outdoor weather [366]. The main conclusions were: (1) SMD II outperformed black powder dusting, being on marks unexposed to water as well as those exposed up to four weeks, (2) the quality of the fingermarks decreased with the length of the exposure to water, but SMD II maintained a non-negligible capacity to detect fingermarks after four weeks of exposure to adverse conditions, and (3) aqueous immersion using stagnant rain water led to larger detrimental impact compared to tap water, a likely explanation being the presence of contaminants and bacteria.

**Miscellaneous** – Considering depletion series of natural fingermarks left of non-porous surfaces and immersed in stagnant water for up to five days, Kapoor et al. obtained good results with two types of dry powders: Robin blue and silver magnetic powder [339]. Using X-ray fluorescence microscopy, Boseley et al. investigated how a detrimental event such as aqueous immersion may impact the inorganic ions contained in fingermarks [150] – See Section 3.1.

Acronyms used: **PE** (polyethylene), **SMD II** (single-metal deposition II), **SPR** (small particle reagent)

#### 4.5.3 Other contextual situations

**Preliminary/Pilot studies** – Fingermarks left on stainless steel metal plates that were buried in the soil for up to eight weeks were successfully detected using SB [378]. The other detection techniques that were considered in this study were black powder, SPR, ORO and CV. Fingermarks left on various non-porous substrates (i.e., finished wood, brass, glass, and plastic) were exposed to simulated outdoor conditions (i.e., temperature and humidity) for up to 30 days before being successfully detected using CTF [447]. These preliminary/pilot studies relying on limited sample sets (e.g., one donor, few depositions, sebum-rich secretions, fresh fingermarks), an overestimation of the reported performances is expected.

**CBRNE threat** – Four papers investigated the impact of (1) blast suppression foam and gel barriers [448], (2) IED neutralization by ballistic water jet [449], (3) decontamination agents [450], and (4) corrosive substances [273] on fingermark recovery. These studies are briefly described here-below, and the most relevant observations/conclusions cited:

- the impact of blast suppression foam and aqueous gel barrier blocks on the recovery of various forensic traces, among which fingermarks, was investigated by Monson et al. [448]. Considering sebum-rich fingermarks left on a variety of substrates (e.g., copy paper, cardboard, electrical tape, duct tape, PVC pipe, metal pipe), the items were exposed to the foam or the gel before being processed with conventional techniques (e.g., RUVIS, [CA ➭ dye staining], WetWop, IND). The authors showed that both the foam and the gel had a significant detrimental impact on fingermark recovery, with no fingermark recovered on nearly all porous and non-porous items, at the exception of metal flats (non-useable ridge details) and adhesives (non-useable ridge details, at the exception of one useable fingermark on the adhesive-side of an electrical tape).

- Vanderheyden et al. asked participants to assemble IEDs composed of eight components (i.e., metal can, PP suitcase, electrical tape, mobile phone, 9V battery, push button, detonator, and circuit board), which were then neutralized by a ballistic water jet or detonated using a small amount of C-4 [449]. The neutralized and detonated IEDs were then processed with [CA ➭ BY40], which led to the detection of 27% and 45% of the fingermarks in presence, respectively. The authors emphasized the fact that the recovery rate associated to the post-blast IEDs may be overestimated due to a low amount of C-4 used (i.e., 7g).
- the impact of CBRNE decontamination agents on the recovery of various forensic traces, among which fingermarks, was investigated by Wilkinson et al. in the frame of an international study involving Australia, Canada and the USA [450]. In the first part of the study, sebum-rich and bloody fingermarks were left on photocopy paper and Ziploc™ plastic bags, and exposed to various decontamination agents (i.e., VHP, gamma irradiation, ozone, dry fogging, formaldehyde, chlorine dioxide, MODEC MDF-500, and Bioxy-S) before being detected using conventional detection techniques (i.e., IND/Zn, DFO, AB, LMG, [CA ➭ BY40], and VMD_Au/Zn_). Gamma irradiation resulted in the least detrimental impact on fingermark recovery, followed by VHP. On the contrary, liquid- and foam-based agents, as well as strong oxidizing decontamination agents (e.g., MODEC MDF-500, Bioxy-S and ozone), had a stronger detrimental impact on fingermark recovery. In the second part of the study, samples bearing fingermarks were first contaminated with *Bacillus thuringiensis* before being decontaminated with gamma irradiation or VHP. However, the samples being contaminated with an aqueous solution containing the bacillus, this protocol had a detrimental impact on the fingermarks before any decontamination step was carried out. Consequently, the results were inconsistent between laboratories and with the first part of the study. Overall, considering all the traces of forensic interest, the authors concluded that the least detrimental decontamination agents were formaldehyde, followed by VHP and gamma irradiation. All three successfully decontaminated samples bearing *Bacillus thuringiensis.*
- the impact of a 5-min-exposure to strong alkali (i.e., up to 4 M potassium hydroxide) and strong acid (i.e., up to 4 M sulfuric acid) on the recovery of fingermarks was investigated by Masterson and Bleay [273]. Considering natural fingermarks left on three substrates (i.e., paper, glass, and PET), the authors showed that fingermarks could still be developed on porous substrates – using PD, and on non-porous substrates – using VMD_Au/Zn_, VMD_Ag_, C-BPS, and Fe-BPS. The other detection techniques that were considered in this study were black magnetic powder, [CA ➭ BY40], SB, ORO, and iodine fuming. The authors also noticed that the exposure to an alkali solution was more detrimental to fingermarks compared to an acid one.

**Cultural heritage** – van der Pal et al. studied how fingermarks could be deposited on paper with regards to different prevention protocols (e.g., glove wearing, hand washing, antibacterial gels) [451]. The authors showed that (1) on the contrary to vinyl and nitrile gloves, secretions could permeate through cotton gloves after an hour of wearing and leave fingermarks (no ridge details), (2) the amount of secretion residue on the skin is higher on washed hands after a couple of minutes compared to unwashed hands, (3) antibacterial gels do not reduce the ability to leave fingermarks and could even increase it (4) incorrect glove hygiene (e.g., touching the face while wearing gloves), results in the deposition of fingermarks (no ridge details).

**Exposure to ethanol –** Park and Hong investigated how an exposure to ethanol may impact fingermark quality and recovery on paper [241]. Considering IND/Zn and ORO as fingermark detection techniques, the authors showed that the exposure to a liquid containing less than 75% (v/v) ethanol (e.g., alcoholic drinks, mouthwash, hand sanitizer) had a detrimental impact on the amino acid fraction. On the contrary, exposure to ethanol >80% (v/v) had a detrimental impact on the lipidic fraction. In case of doubt, the authors recommended processing the item with ORO, for most of the household liquids contain less than 75% (v/v) alcohol. In a second part of the study, the authors showed that contaminating hands with ethanol-containing liquids had no impact on the quality of the deposited fingermarks.

Acronyms used: **AB** (amido black), **BY40** (Basic yellow 40), **C-BPS** (carbon-based black powder suspension), **CA** (cyanoacrylate fuming), **CBRNE** (chemical, biological, radiological, nuclear, and explosive), **CTF** (columnar thin film), **CV** (crystal violet), **DFO** (1,8-diazafluoren-9-one), **Fe-BPS** (iron-oxide-based black powder suspension), **IED** (improvised explosive device), **IND/Zn** (1,2-indanedione/combined with zinc chloride), **LMG** (leucomalachite green), **ORO** (oil red O), **PD** (physical developer), **PET** (polyethylene terephthalate), **PP** (polypropylene), **RUVIS** (reflected UV imaging system), **SB** (Sudan black; solvent black 3), **SPR** (small particle reagent), **UV** (ultraviolet), **VHP** (vaporous hydrogen peroxide), **VMD**_**Ag**_ (silver-based vacuum metal deposition), **VMD**_**Au/Zn**_ (gold/zinc-based vacuum metal deposition)

### 4.6 Miscellaneous

#### 4.6.1 Artificial secretions

**Chemical pads** – The use of commercially-available pads to assess the performance of new detection techniques, or to optimize existing ones, has been reported in several papers, being sweat simulant pads [271] or sebum simulant ones [250,267,269,271]. For Kent, the use of artificial secretions in this context is not recommended, for some chemicals are not present in natural fingermarks and there is no way to know how chemicals may influence the reaction mechanisms [252]. In the same context, Steiner et al. assessed the reliability of such pads [452]. Considering three commercially-available pads (i.e., eccrine, sebum, and mixture), six detection techniques (i.e., IND/Zn, NIN, [CA ➭ R6G], VMD_Au/Zn_, PD), and five substrates (i.e., copy and recycled papers, acetate sheets, glass and glossy paper), the authors showed that: (1) the pads were less prone to qualitative variations, compared to natural fingermarks, (2) the eccrine pads behaved unreliably with several detection techniques (i.e., wrong colour post-NIN, unexpected reaction with PD, persistence post-immersion), (3) conversely, the sebum and mixture pads behaved quite reliably (e.g., colour, reaction products), (4) the exact composition of such pads is sometimes not disclosed by the providers, and (5) the concentration of chemicals in the pads is too high, leading to an over-assessment of the actual detection performances. Consequently, the use of chemical pads to assess the performance of detection techniques is currently not recommended.

**Teaching/Training** – Two papers described the use of artificial secretions to be used in a teaching or training context [453,454]. These studies are briefly described here-below, and the most relevant observations/conclusions cited:

- in a vulgarization paper not dedicated to the forensic community, Azman described the use of a 50:50 (v/v) mixture made of hand lotion (e.g., Vaseline intensive care) and protein powder (e.g., soy protein), to be applied on the fingertip before fingermarks are deposited [453]. The successful reaction with iodine and NIN was illustrated.
- Jeanneret et al. investigated how printed fingermarks could be comparable to natural ones, in terms of luminescence intensity when being processed with luminescent amino acid reagents [454]. Using a chemical printer and dilution series of artificial sweat (i.e., 19 compounds), the authors printed series of artificial fingermarks that were processed with IND/Zn. The measured luminescence intensities were then compared to those obtained with natural fingermarks deposited by a pool of 30 donors. The authors showed that a range of concentrations could cover the intensities observed with fingermarks provided by good to bad donors.

**Quality control** – Four papers referred to the design or assessment of positive control tests for amino acid reagents [[242], [243], [244]] or MS-based techniques [226]. These studies are briefly described here-below, and the most relevant observations/conclusions cited:

- Janssen-Bouwmeester et al.’s control test aimed at assessing if a questioned solution is performant enough to reach the lower LOD determined experimentally [242]. The control tests were printed using a chemical printer and an aqueous solution containing 14 amino acids at a pre-determined concentration (i.e., 0.50 mg/L for IND/Zn and 50.0 mg/L for NIN). In its final version, the control test presented two squares: the first one presenting latent ridge details, the second one being a negative control. The authors observed that the visibility of the control tests decreased over time and was influenced by the surrounding temperature. They hence recommended storing the tests in the freezer (−13.3°C) and using them up to 6 months after printing.
- Croxton et al.’s control test was designed to assess if dilution series of amino acids were comparable to actual fingermarks when trying to assess the performance of a questioned amino acid reagent [244]. The control tests were printed using a refillable inkjet printer and aqueous amino acid solutions (composition and concentrations not disclosed). In its final version, the control test presented dilutions series composed of 16 squares and area for actual fingermarks. Once processed with different NIN formulations (among which improper ones), the control tests were scanned, the density level of each square determined, as well as the fingermarks quality and ridge intensity. The authors concluded that inkjet-printed tests represented an accurate and reliable alternative to fingermarks, to be used as routine amino acid reagent quality controls.
- organized in the frame of the 2017 EFP-WG activities, 51 laboratories conducted a CE during which they were asked to process two control tests with NIN: one bearing dilution series of printed patterns, and one bearing depletion series of natural fingermarks [243]. The control tests were printed using a modified inkjet printer and an aqueous solution containing 10 amino acids. In their paper, the authors detailed the way the CE was created, how the results were assessed, as well as a detailed analysis of the gathered results. The main conclusions were: (1) NIN is a robust process as it can overcome the methodological differences observed between laboratories, and (2) printed test strips represent a more reproducible way of assessing the performance of a questioned solution compared to actual fingermarks.
- Gorka et al.’s control test was designed to be compatible with MS imaging techniques, such as MALDI-MSI [226]. The proposed template combined geometric patterns (i.e., full and lined squares) with a complex chemical composition made of eccrine-based and sebum-based compounds. The authors used a chemical printer to print both kinds of artificial secretions sequentially, in a multi-layer approach, and demonstrated its successful application to MALDI-MSI, NIN, and ORO. Further experiments are expected, to address some issues encountered with the chemical printer.

**Miscellaneous** – Steiner et al. investigated the preparation and deposition of an emulsion made of artificial sweat and sebum, as well as its reactivity with four detection techniques: [IND/Zn ➭ NIN ➭ ORO ➭ PD] [455]. Promising results were obtained with regards to IND/Zn, NIN, and ORO; however, unreliable behaviour was observed with PD. To assess DNA and protein recovery techniques, LeSassier et al. described a suspension made of eccrine sweat and sweat:sebum emulsion (commercially available), extracellular DNA standard (commercially available), and epidermal skin material (collected from donors) [456]. Promising results were obtained with regards to DNA and protein recovery, but the application of fingermark detection techniques was not tested, for it was not the primarily objective of this study.

Acronyms used: **CA** (cyanoacrylate fuming), **CE** (collaborative exercise), **EFP-WG** (ENFSI Fingerprint working group), **IND/Zn** (1,2-indanedione combined with zinc chloride), **LOD** (limit of detection), **MALDI** (matrix-assisted laser desorption ionization), **MS/I** (mass spectrometry/imaging), **NIN** (ninhydrin), **ORO** (oil red O), **PD** (physical developer), **R6G** (rhodamine 6G), **VMD**_**Au/Zn**_ (gold/zinc-based vacuum metal deposition)

#### 4.6.2 Touch DNA

The aim of this report was not to extensively cover the question of genetic material contained in secretion residue (i.e., touch DNA). Only the studies addressing touch DNA in relation with fingermark detection techniques were reported below.

**Preliminary/Pilot studies** – Harush-Brosh et al. proposed to lift the fingermarks at crime scene with gelatine gel lifters, that would then be processed in the laboratory for fingermarks, using BPS, or for DNA profiling [457,458]. Promising results were obtained for fingermark detection with glass and polymer-coated metal car parts, but the approach failed to provide results on plastered wall. To detect fingermarks and emphasize the presence of cellular material, Khuu et al. proposed to simultaneously apply IND/Zn and DMAB on paper [459]. Promising results were obtained, but the DNA profiling must be conducted the same day as the detection to avoid DNA degradation over time. The authors also emphasized that cells tend to be found on the sides of fingermarks, rather than in their centre. Nontiapirom et al. showed that genetic material could be present on new fingerprint brushes, and that DNA transfer could occur if the brush is applied on a fresh/wet spot (e.g., saliva) before dusting fingermarks [335]. CTF was shown to be compatible with DNA profiling when applied on bloody fingermarks [460] or on sebum-rich fingermarks exposed to different environmental conditions [461]. The Hussa portable CA device seems to have no detrimental impact on DNA [462]. Most of these preliminary/pilot studies relying on limited sample sets (e.g., one donor, few depositions, sebum-rich secretions, fresh fingermarks), an overestimation of the reported performances is expected.

**[Fingermarks ➭ DNA]** – Four papers investigated the impact of fingermark detection techniques on DNA profiling: IND/Zn, DFO, NIN, ORO and PD [463], IND/Zn and DFO on cigarette butts [464], dusted and lifted fingermarks [465,466]. These studies are briefly described here-below, and the most relevant observations/conclusions cited:

- on copy paper, Bathrick et al. determined that IND/Zn was the least harmful technique, followed by NIN and ORO [463]. On the contrary, DFO and PD were the most detrimental processes. The authors also recommended limiting the number of techniques if DNA analysis is planned.
- on cigarette butts collected among smokers and processed with IND/Zn or DFO, Lee et al. observed a drop in DNA quantity equal to −27% and −16%, respectively, compared to unprocessed cigarette papers [464]. IND/Zn seemed to have a more detrimental impact on DNA profiling compared to DFO. It should be noted that both IND/Zn- and DFO-processed items were exposed to 180°C for 10 s, which is an unconventional protocol.
- Romano et al. and Menchhoff et al. confirmed the possibility to obtain DNA profiling from fingermarks that have been dusted and stored on latent fingerprint lift cards for years [465,466].

**[DNA ➭ Fingermarks]** – Two papers investigated the impact of DNA recovery on the detection of fingermarks: use of a gelatine gel lifter on porous and non-porous items [467], use of a dry vacuum technique [468]. These studies are briefly described here-below, and the most relevant observations/conclusions cited:

- during a pseudo-operation trial encompassing various porous and non-porous item, Fieldhouse et al. used gelatine gel lifters to collect DNA before fingermark detection techniques were applied [467]. The authors concluded that (1) useable fingermarks could still be detected after the collection of DNA, (2) substrate roughness played a major role on the quality of the recovered fingermarks. Note – The fingermarks on non-porous items having not been observed beforehand, through visual examination, it was not possible to actually assess the quality drop induced to fingermarks by the collection of DNA.
- in a preliminary study, McLaughlin et al. presented a new way to collection DNA, based on the swabbing of a porous item using a swab inserted in a glass pipette linked to a vacuum pump [468]. Promising results were obtained, for the recovery of DNA induced no detrimental impact on the idented text or the fingermarks in presence.

**Visualisation of touch DNA** – Several papers referred to Diamond™ Nucleic Acid Dye to emphasize the genetic material contained in fingermarks [[469], [470], [471], [472], [473], [474], [475], [476]]. Some ridge details could be observed, but this approach was mostly presented to emphasize areas of interest or investigate shedder status. These papers were hence cited but not further detailed in this report.

**Other sections** – The following papers were detailed in another section of this report: processing of stamps for fingermarks and DNA [437] – See Section 4.4.6; use of artificial fingerprint [456] – See Section 4.6.1.

Acronyms used: **BPS** (black powder suspension), **CA** (cyanoacrylate fuming), **CTF** (columnar thin film), **DFO** (1,8-diazafluoren-9-one), **DMAB** (p-dimethylaminobenzaldehyde), **IND/Zn** (1,2-indanedione combined with zinc chloride), **NIN** (ninhydrin), **ORO** (oil red O), **PD** (physical developer)

#### 4.6.3 Other

**Fingermark grading** – A preliminary study proposed to use densitometric image analysis as a complementary grading method when split marks are considered [477]. The following three papers investigated the grading of fingermark quality in a detection context: impact of native images and terms referring to the suitability for identification on the decisions taken by evaluators [478], automatic assessment of fingermark quality [68], and guidelines for a correct use of ordinal data generated by fingermark quality grading scales [479]. These studies are briefly described here-below, and the most relevant observations/conclusions cited:

- Barnes et al. asked evaluators to grade the quality of a set of detected fingermarks by considering two scales [478]. Both scales were similar regarding ridge detail descriptions, but one included terms linked to the suitability for identification. The authors also asked the evaluators to grade native (i.e., coloured) and greyscale (i.e., dark ridges on light background) images of the same set of fingermarks. The main conclusions were: (1) although similar results were obtained overall, a shift towards higher scores was observed in favour of the grading scale that included identification-related terms, (2) a shift towards higher scores was observed in favour of the native images for NIN and [DFO ➭ NIN], (3) the opposite trend was observed for fingermarks detected by black powder. Dove reacted to this paper, for the grading scale referring to the suitability for identification was used in a study he conducted recently [480].
- Bonnaz et al. investigated how quality assessment algorithms applied in an identification context could be used to assess large sets of fingermarks, such as those generated during the development or optimization of a fingermark detection technique [68]. Natural fingermarks were left on three substrates (i.e., office paper, PP sleeves, white ceramic tiles), processed with two conventional techniques (i.e., CA and IND/Zn), and graded by five human evaluators and seven algorithms. The authors determined that LQM was the most promising algorithm, for it agreed with the grading provided by the human evaluators. The other tested algorithms were Lights Out, LFIQ 1 and 2, ESLR, NIST FIQ, and MINDTCT. Further studies are expected on this topic.
- Hockey et al. provided guidance about the use of the Home Office fingermark grading scale, or of any scale generating ordinal data [479]. In this context, the authors recommended displaying the results as frequency tables or bar graphs, rather than averaging the scores. For those willing to present statistical tests, the authors presented some parametric (e.g., difference of proportion) and non-parametric (e.g., Kruskal-Wallis, Mann-Whitney U, Chi-squared, or Wilcoxon signed-rank) tests that would suit the Home Office grading scale. Finally, the authors provided some hints about how to graphically represent the results when comparing different sequences.

**Surface interaction** – Hughes et al. investigated the affinity of non-porous substrates towards saliva and fingermarks [481]. The authors considered natural fingermarks left on non-porous substrates presenting different degrees of roughness, which were characterized by Raman spectroscopy, AFM, FE-SEM, and water contact angle). The parameters impacting the deposition of fingermarks were identified and discussed, among which: surface roughness, hydrophobicity, surface free energy, and composition of the deposited material. In a similar context, but not directly linked to the forensic field, the following paper may be of interest to some researchers as it discussed the development of “anti-fingerprint” glass [482].

**Operational performance** – Koning reported the recovery rates associated to 2′000 cases processed in 2017 and 2018 by the Colorado Bureau of Investigation – Forensic Services laboratories (i.e., 500 cases per laboratory) [426]. The sequences associated to porous and non-porous items were [visual examination ➭ IND/Zn or DFO ➭ NIN] and [visual examination ➭ CA ➭ dye], respectively. The recovery rates were determined by dividing the number of items bearing at least one fingermark of value by the total number of processed items. The items were dispatched among ten categories and extensive data was provided in the paper. The highest recovery rates were associated to porous items (e.g., paper checks - 66.67%, regular paper items - 46.56%, and envelopes - 32.47%) and the lowest to cartridge cases (i.e., 0.38%). Common non-porous substrates resulted in good recovery rates (e.g., plastic bottles and cups - 23.45%, aluminium cans - 28.64%). The recovery rates associated to firearm-related items can be found in Section 4.4.4. Following this study, data-driven decisions were taken: some types of evidence are no longer accepted routinely for fingermark detection (e.g., cartridges, rocks, raw wood, syringes), and some procedures were revised (e.g., tools and drug-related plastic wraps).

Acronyms used: **AFM** (atomic force microscopy), **CA** (cyanoacrylate fuming), **DFO** (1,8-diazafluoren-9-one), **FE-SEM** (field-emission scanning electron microscopy), **IND/Zn** (1,2-indanedione combined with zinc chloride), **LFIQ** (Latent Fingerprint Image Quality), **LQM** (Latent Quality Metric), **MINDTCT** (the minutiae detector from the NIST Biometric Image Software - NBIS), **NIN** (ninhydrin), **NIST FIQ** (National Institute of Standards & Technology NIST Fingerprint Image Quality), **PP** (polypropylene).

## 5 Other body marks, case reports and miscellaneous

This last section of our report covers various methods of personal identification based on marks and impressions and a few case studies without the ambition of exhaustiveness.

### 5.1 Lip marks and lip prints

Constantly portrayed in the literature as unique for any individual, lip prints received limited research attention and certainly not to the level in line with the bold claim of uniqueness. As rightly indicated by Fronseca [483] in their critical review: “Currently, authors continue to use the concept of lip pattern uniqueness, yet the greatest part of their research has failed to support this hypothesis under current scientific standards” and later “Lip print identification has been important historically, but the new paradigm makes the redefinition of the current research necessary to stop guesswork and speculation”. Their analysis is without compromise and shared by the authors of this report.

Most of the published research is from India. Kaur and Thakar [484] proposed a new classification system (7 main patterns and 43 combinations) allowing the consideration of partial impressions (the whole impression divided in 10 quadrants) and tested it on lip prints taken from 500 individuals. One study [485] indicated their ability by visual comparison to distinguish marks and prints from 102 subjects (52 males and 50 females). The research in the future may benefit more from machine learning techniques developed for in biometric applications [486]. However, at the moment, efforts are more devoted to lip images as a visible external attribute than on marks or prints left on surfaces. One research group in Poland published regularly on the lip print biometrics with regularly improved performance [487]. They reported an accuracy above 94% on a database of 1001 lip prints taken from 143 individuals (7 lip prints per person) with a technique allowing to detect low quality areas in the prints. Sandhya et al. [488] obtained an accuracy of 97% on a smaller dataset (300 individuals) using an ensemble classifiers informed by three classifiers (KNN, SVM, ANN).

### 5.2 Bite marks

The excellent recent book by Fabricant [489] brought back to the front a number of cases where bitemark evidence have been used with a strength that is not deserved. In some cases, subsequent DNA analyses have helped exposing misattributions based on bitemark attributions. For the present report we limit ourselves to refer to the review work of the NIST [490]. Based on the analysis of over 400 sources, they concluded that: “Based on this input, our study found a lack of support for three key premises of the field: 1) human dentition is unique at the individual level, 2) this uniqueness can be accurately transferred to human skin, and 3) identifying characteristics can be accurately captured and interpreted by analysis techniques.”

We see no other avenues that to invest in systematic research taking advantage of computer-assisted methods as suggested by Ref. [491].

### 5.3 Ear marks

During this reviewing period, few research have been devoted to ear marks. Studies have explored the variability of the external ear based on photographs [492], but less work tried to address the issue of the marks left on scenes. In this field also deep learning techniques have proved their potential [493] with an accuracy of 97%. That being said, it is important to observe that the study was conducted on images of the ear. It is closer to ear biometry [494] or external ear studies [495,496] than the forensic use of earmarks. However, as we will present later with a few fingerprint cases, images of ears from individual of interest may become a new source of trace images submitted to forensic laboratories because of the larger availability of CCTV images or images published on social media.

### 5.4 Case studies

A case where two marks with very similar appearance (set of ridges and background noise) from the same portion of friction ridge skin is presented in Ref. [27] to show that such instances may occur without any attempt of forgeries or fabrication.

Social media provide a rich source of images that show friction ridge skin that can be potentially used for identification. Grilli [497] presents a case of an association established based on an image of a hand handling a gun. It is the friction ridge skin details visible in fingers and phalanges that have been compared against the 10-print card of the individual of interest. Another case is presented by Forsyth [498] where friction ridge skin images from the side of the fingers had been used to identify two individuals subsequently convicted as paedophiles.

Patent fingermarks are often found in conjunction with archaeological artefacts [14,15,[499], [500], [501]]. Their investigation helps to reconstruct social and work activities.

The French gendarmerie published the technical details of the fingerprint Disaster Victim Identification (DVI) toolkit used by their specialists to attend large-scale disasters [502]. It includes traditional fingerprint recovery and restructuring techniques, but also includes a livescan device and a portable AFIS system to speed up comparison with ante-mortem material. The livescan device has been selected among three competitors using real corpses.

The recovery of prints from mummified body recovered from a salt pond has been presented by Poux et al. [503]. The fingers were immersed in soapy water (a few drops of dishwashing liquid in water) for 20 h. The epidermis was removed and suitable prints were obtained from the dermis following an injection of a restructuring gel underneath. On a nearly 300 years old mummy, three-dimensional scanning and close-range photogrammetry have been used by Urbanová et al. [504] followed by two techniques to stretch, flatten, and successfully map the three-dimensional image into a two-dimensional record. The techniques for unrolling – representing a 3D surface as a 2D image – will play a significant role both for post-mortem fingerprint recovery [505] and for livescan on-the-fly scanning.

### 5.5 Miscellaneous

We note the development of a specific scale to be used to record marks from scene that is coupled with dedicated software allowing the production of 1:1 scale images including a correction deviation from a perpendicular angle (up to 20°) [506].

## Footnotes

1 https://www.justice.gov/olp/uniform-language-testimony-and-reports.

2 https://www.aafs.org/sites/default/files/media/documents/013_Std_Ballot03.pdf.
